# Polycystic ovary syndrome in obstructive sleep apnea-hypopnea syndrome: an updated meta-analysis

**DOI:** 10.3389/fendo.2024.1418933

**Published:** 2024-08-23

**Authors:** Jie He, Xia Ruan, Jia Li

**Affiliations:** ^1^ Clinical Medical College of Chengdu Medical College, Chengdu, Sichuan, China; ^2^ Department of Pulmonary and Critical Care Medicine, The First Affiliated Hospital of Chengdu Medical College, Chengdu, Sichuan, China; ^3^ Key Laboratory of Geriatric Respiratory Diseases of Sichuan Higher Education Institutes, Chengdu, Sichuan, China; ^4^ Department of Rehabilitation, The First Affiliated Hospital of Chengdu Medical College, Chengdu, Sichuan, China

**Keywords:** PCOS, OSAHS, prevalence, metabolism, meta-analysis

## Abstract

**Background:**

Obstructive sleep apnea-hypopnea syndrome (OSAHS) is correlated with metabolic deterioration in patients experiencing polycystic ovary syndrome (PCOS). Women diagnosed with PCOS exhibit a heightened prevalence of OSAHS. This meta-analysis aims to assess the morbidity of OSAHS in women affected by PCOS and to examine the differences in metabolism-related indicators between OSAHS-positive and OSAHS-negative in women with PCOS.

**Methods:**

A comprehensive literature analysis of OSAHS morbidity in women with PCOS was conducted, utilizing databases such as CNKI, EMBASE, PubMed, Web of Science, and Wanfang. A comparison was carried out between patients with OSAHS-positive and those with OSAHS-negative in terms of their clinical characteristics and metabolic differences. The search language included English and Chinese. The acquired data were analyzed by employing RevMan 5.2 and Stata 11.0. Continuous variables with the same units were combined and analyzed through weighted mean differences (WMDs) as effect sizes, while continuous variables with different units were combined and analyzed through standardized mean differences (SMDs) as effect sizes. A conjoint analysis was performed on the basis of I^2^ value, using either a fixed effect model (I^2^ ≤ 50%) or a random effect model (I^2^ > 50%).

**Results:**

A total of 21 articles met the inclusion criteria for this study. The findings indicated that 20.8% of women with PCOS were found to have comorbid OSAHS. The subjects were categorized into various subgroups for meta-analysis on the basis of race, age, disease severity, body mass index (BMI), and diagnostic criteria of PCOS. The results revealed high morbidity of OSAHS in all subgroups. In addition, most metabolic indicators and parameters of metabolic syndrome were notably worse in women suffering from both PCOS and OSAHS in comparison to their counterparts solely diagnosed with PCOS.

**Conclusion:**

The current literature indicates higher morbidity of OSAHS among women with PCOS, linking OSAHS with worse metabolic status and obesity in this population. Consequently, clinicians are advised to prioritize the detection and management of OSAHS in women with PCOS.

**Systematic Review Registration:**

https://www.crd.york.ac.uk/PROSPERO/#myprospero PROSPERO, identifier (CRD42024528264).

## 1 Introduction

Polycystic ovary syndrome (PCOS) is a widespread endocrine disorder in women of childbearing age, impacting approximately one out of every ten women (1). It is a metabolic syndrome, presenting with clinical manifestations such as ovulatory dysfunction, hyperandrogenism, and polycystic ovaries (2). Although the exact cause of Polycystic Ovary Syndrome (PCOS) remains unclear, it is likely multifactorial. Insulin resistance (IR) and hyperandrogenism are the two main hormonal disturbances in PCOS. Obesity, genetics, lifestyle, and environmental factors also contribute to the multifactorial etiology of PCOS (3–7). Women with PCOS may experience numerous reproductive, metabolic, psychological, and anthropometric complications (8–10). The most common characteristic of PCOS is ovarian dysfunction caused by hyperandrogenism, leading to chronic oligo-ovulation/anovulation and menstrual irregularities (11–13). Infertility in women with PCOS is the most common cause of anovulatory infertility, but not all women with PCOS are infertile. There are, however, some risks associated with pregnancy and foetal complications regardless of the mode of conception for those who become pregnant (14, 15). Furthermore, affected women are more likely to experience moderate to severe depression and anxiety symptoms compared to healthy women (16). PCOS has metabolic effects, including insulin resistance (IR), dyslipidemia, and abnormal glucose metabolism (17). Additionally, women with PCOS show a tendency toward excessive weight gain, which exacerbates these symptoms (18). Accompanied by increased cardiovascular risk factors such as chronic inflammation, oxidative stress, and impaired fibrinolysis, there is evidence suggesting a higher prevalence of cardiovascular disease in PCOS patients (19).

Obstructive sleep apnea-hypopnea syndrome (OSAHS) is a common medical condition that affects not only women with PCOS and obesity but also non-PCOS women. (20, 21). The clinical presentation of OSAHS in women differs from that in men and may vary with age and physiological status (such as menopause and pregnancy) (22). Overall, compared to men, women seem to have more symptoms of OSAHS, with lower Apnea-Hypopnea Index scores. Furthermore, they appear to have longer periods of partial upper airway obstruction, and may more frequently report insomnia as a symptom of OSAHS (23). It is marked by recurrent episodes of decreased oxygen saturation, sleep fragmentation, and upper airway occlusion, followed by aberrant secretion of gonadotropin-releasing hormone, increased sympathetic activity, insulin resistance (IR), and oxidative stress (OS) (24). These hormonal imbalances and metabolic irregularities are also commonly observed in PCOS and are believed to contribute to its etiology. Thus some researchers argue that OSAHS may exacerbate the severity of PCOS in affected women (25).

Numerous studies have investigated the association between PCOS and OSAHS. However, the available evidence presents some contradictions. A meta-analysis conducted by Kahal et al. (26) demonstrated no considerable difference in total testosterone (TT) and free testosterone (FT) levels between women diagnosed with both PCOS and OSAHS in comparison to those with PCOS alone. Nevertheless, Fogel et al. (27) reported that TT and FT were positively correlated with the apnea-hypopnea index (AHI). In addition, certain studies have indicated a heightened prevalence of OSAHS among women with PCOS (11, 27). This is further supported by the *Endocrine Society Clinical Practice Guideline* (28), which in its analysis of the evidence states that “the rate of OSAHS in women with PCOS equals or exceeds that in men.” Nevertheless, there is a wide range of reported rates of OSAHS in women diagnosed with PCOS, ranging from 0% to 70% (12, 29), potentially influenced by the sample size of the study.

Therefore, this study summarizes the existing published literature related to PCOS and OSAHS and conducts a comprehensive analysis of the morbidity of OSAHS in women affected by PCOS. Moreover, a systematic review and meta-analysis are performed to explore the correlation between OSAHS and metabolic irregularities in women affected by PCOS. Furthermore, apart from weight loss, women with both PCOS and obesity have limited options for safe and effective treatment. Research has demonstrated that continuous positive airway pressure (CPAP) can ameliorate IR, mitigate OS and inflammatory status, and enhance the quality of life in patients diagnosed with OSAHS. Thus, investigating the effect of OSAHS on women suffering from PCOS may contribute to the development of new therapeutic strategies for this population.

## 2 Methods

The systematic evaluation methodology used in this study has been registered prospectively at PROSPERO (https://www.crd.york.ac.uk/PROSPERO/#myprospero, ID: CRD42024528264). The findings of this study are reported as per the Preferred Reporting Items for Systematic Reviews and Meta-Analyses (PRISMA) guidelines.

### 2.1 Inclusion and exclusion criteria

This systematic review and meta-analysis will include cross-sectional, cohort, case–control studies and randomized control trials. These studies were designed to detect whether women with PCOS had OSAHS. The included studies utilized polysomnography (PSG) or class III devices for diagnosing OSAHS. Subjects met the diagnostic criteria for OSAHS based on PSG or class III devices (adults: AHI≥5/h; children: AHI≥1/h) (30).

The study encompassed women with PCOS, irrespective of age (adolescents-postmenarcheal and adults-postmenopausal and premenopausal), race, or PCOS diagnostic criteria. Polycystic ovary syndrome was diagnosed according to the revised 2003 Rotterdam criteria (31). Several studies reported that the diagnosis of polycystic ovary syndrome broadly followed the consensus criteria from the National Institutes of Health of anovulation and hyperandrogenaemia (32).

The exclusion criteria encompassed conditions with a similar phenotype to PCOS, such as androgen-secreting tumors, Cushing syndrome, prolactinoma, congenital adrenal hyperplasia, and thyroid diseases. Abstracts, letters to the editor, reference papers (most conference papers only have abstracts instead of complete data), animal experiments, and case studies were also excluded from this study.

### 2.2 Main results

Morbidity of OSAHS in women with PCOS.

### 2.3 Secondary results

Morbidity of OSAHS in women with PCOS in comparison to women without PCOS.

Differences between women diagnosed with both PCOS and OSAHS and women with PCOS alone regarding blood lipid, blood pressure (BP), body mass index (BMI), blood glucose, hemoglobin (HGB), hypertrichosis, waistline, insulin (INS), IR, low-density lipoprotein (LDL), waist-to-hip ratio (WHR), two-hour oral glucose tolerance test (2-h OGTT), TT, FT, sex hormone-binding globulin (SHBG), etc.

### 2.4 Search methodology

Searches were conducted across CNKI, EMBASE, PubMed, Web of Science, and Wanfang databases for non-English and English articles. The keywords and subject terms used were “polycystic ovary syndrome” or “ovary polycystic disease” or “PCOS” or “stein leventhal” or “hyperandrogenic anovulation” and “Obstructive Sleep Apnea-Hypopnea Syndrome” or “Obstructive Sleep Apnea” or “Obstructive Sleep Apnea Syndrome” or “OSA” or “OSAHS” or “OSAS”. The retrieval spanned from the inception of the databases up to March 1, 2024. In addition to the computerized search, a manual search was conducted on all retrieved articles. Articles with potential relevance were assessed according to pre-specified inclusion and exclusion criteria.

### 2.5 Literature selection

Two authors (He J and Ruan X) independently screened the title and abstract of each article. All articles with potential relevance were thoroughly reviewed. Any differences between the two authors were resolved through consensus and, if necessary, discussed with a third author (Li J).

### 2.6 Data extraction and management

Data extraction was independently conducted by two authors (He J and Ruan X). In the case of multiple publications, the primary study or paper was included, with **Supplementary Information** extracted from secondary papers where necessary. As needed, the authors of the papers were contacted to address data queries and resolve disputes regarding the data. In case of duplicate publications, the original authors were contacted to clarify the main publication. If no response was received, the study with the highest number of participants was selected. Details that were extracted from each study included participant characteristics, study design, and morbidity data.

### 2.7 Assessing risk of bias

Quality assessment was carried out independently by two reviewers (He J and Ruan X) and any differences were resolved through negotiation and discussion with the third reviewer (Li J). The non-randomized Study Bias Risk Assessment Tool (RoBANS) (33) was used to assess the risk of bias in each included study, assessing the following areas of bias risk: selection bias (sample population), selection bias (confounding variables), performance bias (exposure measures), performance bias (analytical methods to control for bias), and other biases. Each area is classified as high risk; low risk; or not clear.

### 2.8 Statistical analyses

We pooled prevalence estimates using the DerSimonian-Laird random-effects model (34). We assessed inter-study differences in prevalence estimates using Higgins I^2^ statistics, with values more than 50% being considered moderately heterogeneous according to recommendations (35, 36). We performed a series of meta-regressions and subgroup analyses to explore the effect of covariates on prevalence estimates. The following factors were considered: race, age, BMI (obese and non-obese), and PCOS definition. For continuous variables, fixed-effect model analyses combined statistically homogeneous findings. Otherwise, we used random-effects meta-analysis. The (weighted) mean difference and SD were calculated to describe the continuous outcomes that were measured on the same scale. The SMD and SD were used to describe continuous outcomes that were not measured on the same scale. Inter-study heterogeneity was assessed using Higgins’s I^2^ statistic, with values more than 50% indicating moderate heterogeneity. All analyses were performed in Stata version 11 for Windows (Stata Corp, College Station, Texas).

## 3 Results

### 3.1 Search results and study attributes

**Figure 1:**
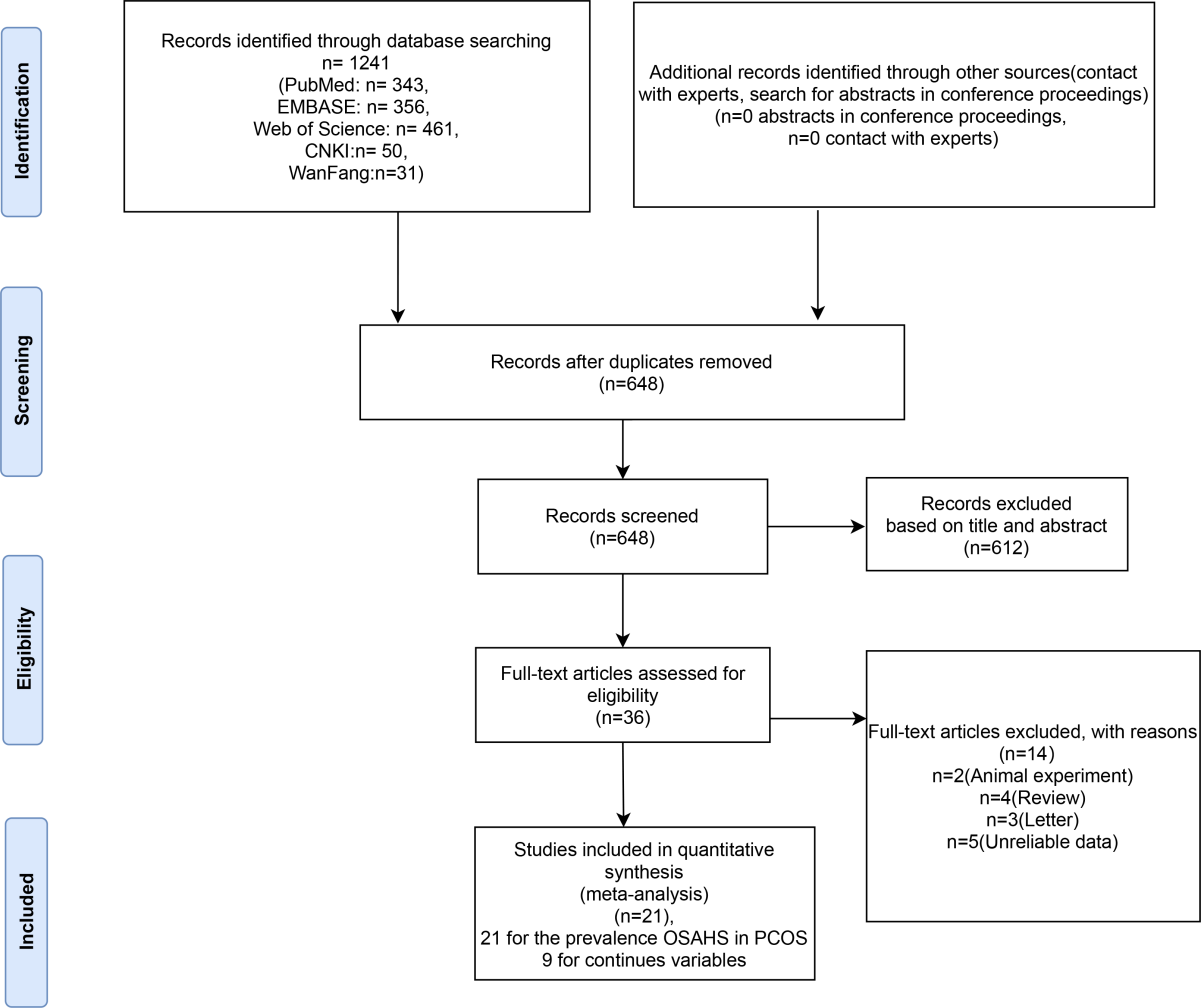
Flow chart of inclusion criteria in the study.

A total of 1241 relevant studies were retrieved from the database. Removing duplicate studies by reading abstracts and titles; 612 articles were eliminated, leaving 38. We downloaded the 36 articles and read through them, excluding 14 papers based on inclusion and exclusion criteria. The articles were excluded for the following reasons: two were animal experiments, four were reviews, three were letters to the editor, and five lacked relevant data. We identified 21 studies (11, 12, 26, 27, 29, 37–57) involving OSAHS incidence in PCOS patients, as shown in **Table 1**. There are 9 literature (11, 37, 39, 41, 43, 50, 53, 56, 57) comparing the clinical characteristics of PCOS patients with OSAHS positive and OSAHS negative (**Table 1**). The flow chart of systematic reviews and meta-analyses for the selection and screening of articles is shown in **Figure 1**. The basic information of the studies is summarized in **Table 1**.

**Table 1:** Characteristics of included studies.

Author	Year	Country	Study design	N		Ethnicity	Age (year)	BMI (kg/m^2^)	PCOS	OSAHS	Outcomes
				Case	Control						
Fogel RB	2001	USA	CSS	18	18	Caucasian	31.1 ± 5.5	36.9 ± 5.5	NIH	PSG	①②④⑯
Vgontzas AN	2001	USA	CSS	53	452	Caucasian	30.4 ± 6.6	38.7 ± 8.0	NIH	PSG	①②⑪⑮⑯
Gopal M	2002	USA	CSS	23	NR	Caucasian	NR	42.5 ± 8.5	NR	PSG	①②
Tasali E	2008	USA	CSS	52	21	Caucasian	29.7 ± 5.1	39.2 ± 7.2	NIH	PSG	①②⑧⑪⑫⑬⑭⑮⑯
Yang HP	2009	China	CSS	18	10	Asian	29.1 ± 6.1	21.7 ± 2.4	Rotterdam	PSG	①②③④⑥⑦⑧⑨⑪⑭⑮
de Sousa G	2012	Germany	CSS	35	19	Caucasian	15.3 ± 1.2	32.9 ± 6.4	NIH	PSG	①②③⑧⑨⑪⑫⑭⑮
Nandalike K	2012	USA	CSS	28	56	Mixed	16.8 ± 1.9	44.8 ± 8.8	Rotterdam	PSG	①②⑤⑥⑨⑩⑪⑭⑮⑱⑲
Mokhlesi B	2013	USA	CSS	44	34	Caucasian	27 ± 5	35.1 ± 11.4	NIH	PSG	①②③④⑧⑪⑭⑮
Chatterjee B	2014	India	CSS	50	NR	Caucasian	NR	28 ± 3.0	Rotterdam	PSG	①②③⑥⑦⑧⑩⑪⑭⑮⑯⑰⑱⑲
Sirmans SM	2014	USA	CSS	1689	5067	Mixed	25.24	>30	NR	NR	①
Tock L	2014	Brazil	CSS	38	NR	Caucasian	28.3 ± 6.8	32.9 ± 7.7	Rotterdam	PSG	①②③④⑧⑨⑩⑪⑫⑭⑯⑰⑱⑲
Lin TY	2017	China	CSS	4595	4595	Asian	28.0 ± 6.79	NR	NR	NR	①
Kumarendra B	2019	UK	CS	76978	143077	Caucasian	30.2 ± 7.4	28.6 ± 7.6	NR	NR	①②
Torres-Zegarra C	2021	USA	CS	92	NR	Mixed	15.4 ± 2.3	35.5 ± 6.6	NR	NR	①②⑨⑬⑱⑲
Zhou X	2021	USA	CSS	200	NR	Mixed	28.0 ± 6.2	30.9 ± 9.0	Rotterdam	BQ	①②③⑥⑨⑩⑪⑭⑮⑱⑲
Underland LJ (obesity)	2022	USA	CSS	21	8	Caucasian	16.38 ± 2.4	38.4 ± 2.5	NR	PSG	①②⑤⑧⑩⑭⑯⑰⑱⑲
Underland LJ (non-obesity)	2022	USA	CSS	10	8	Caucasian	17.50 ± 1.8	23.0 ± 1.8	NR	PSG	①②⑤⑧⑩⑭⑯⑰⑱⑲
Yang R (Mild)	2022	China	CSS	328	NR	Asian	29.2 ± 3.9	28.4 ± 3.7	Rotterdam	PSG	①②⑥⑫⑬⑯⑰⑱⑲
Yang R (Moderate)	2022	China	CSS	328	NR	Asian	30.0 ± 4.0	31.7 ± 4.9	Rotterdam	PSG	①②⑥⑫⑬⑯⑰⑱⑲
Yang R (Severe)	2022	China	CSS	328	NR	Asian	33.5 ± 3.6	32.1 ± 3.3	Rotterdam	PSG	①②⑥⑫⑬⑯⑰⑱⑲
Ibrahim S	2023	USA	CSS	48	NR	Caucasian	NR	NR	NR	BQ	①②⑥
Zhang Q	2024	China	CS	156	NR	Asian	30.1 ± 3.5	23.9 ± 3.6	Rotterdam	PSG	①②⑨⑪
Christ JP	2024	USA	CSS	309	NR	Caucasian	18-44	38 ± 9.1	Rotterdam	BQ	①②③⑧⑨⑩⑪⑫⑬⑭⑮⑯⑰⑱⑲

CSS, cross-sectional study; CS, cohort study; NR, Not reported; BQ, Berlin Questionnaire; PSG, Polysomnography; NIH, National Institutes of Health; BMI, body mass index. ①: Prevalence; ② BMI; ③: Waist circumference; ④: Waist-hip-ratio (WHR); ⑤: BMI Z-score; ⑥: Blood pressure; ⑦: Modified Ferriman-Gallwey score for Hirsutism; ⑧: Sex hormone binding globulin (SHBG); ⑨: Total Testosterone; ⑩: Free Testosterone; ⑪: Fasting plasma glucose; ⑫: 2-hour plasma glucose on OGTT; ⑬: Hemoglobin A1c; ⑭: lnsulin resistance (HOMA-IR); ⑮: Fasting Insulin; ⑯: Total cholesterol; ⑰: Low density lipoprotein(LDL); ⑱: Triglycerides; ⑲: High density lipoprotein(HDL); PCOS, polycystic ovary syndrome; OSAHS, obstructive sleep apnea-hypopnea syndrome.

### 3.2 Risk of bias for inclusion of studies

**Supplementary Figure 1** lists the risk assessment of bias for each study. 9 studies had high selection bias due to inadequate selection of subjects, 2 studies had low risk, and the remaining 13 studies were unclear. Selection bias due to inadequate identification and consideration of confounding variables was high in most studies (14 of 24), low in only 1 study, and unclear in the remaining 9 studies. Performance bias due to inadequate exposure measurements was low in 19 studies, high in 4 studies, and unclear in the remaining 1 study. Most studies (10 of the 24 studies) had low detection bias due to inadequate blindness in outcome assessment, and only 3 studies had high detection bias. Loss of follow-up bias due to mishandling of incomplete outcome data was low across all 24 studies. Most studies (18 out of 24) had low reporting bias due to selective reporting.

### 3.3 Prevalence of OSAHS in women diagnosed with PCOS

**Figure 2:**
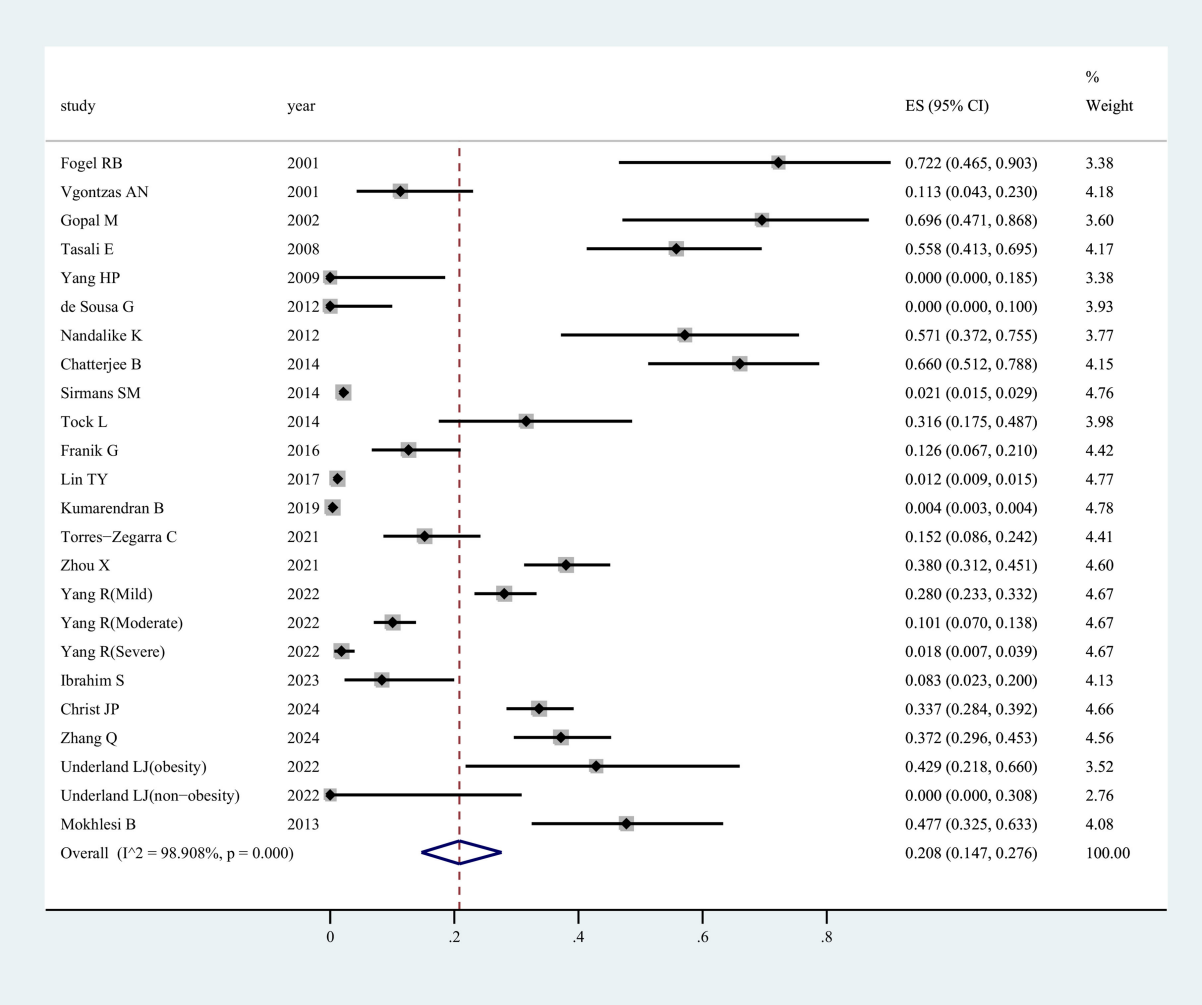
Prevalence of OSAHS in patients with PCOS as determined by the random efforts model.

**Figure 2** presents the prevalence of OSAHS in women affected by PCOS. The morbidity of OSAHS in all studies was about 20.8% [95% confidence interval (CI): 14.7 – 27.6%]. The I^2^ value of 98% indicated statistically significant heterogeneity among the studies. **Supplementary Figure 1** displays the contour-enhanced funnel plot for the publication bias test. Evidence of publication bias was found in the meta-analysis, and the funnel plot showed asymmetry. This was confirmed through a formal test using Egger’s regression test (*P*=0.013) (**Supplementary Figure 2**). Sensitivity analysis revealed that the exclusion of any individual study from the analysis did not undermine the overall findings of the combined analysis.

### 3.4 Subgroup analysis

Evidence suggests that factors, such as disease severity, BMI, race, age, and diagnostic criteria, influence the morbidity of OSAHS. In a subgroup analysis based on disease severity, it was observed that women with PCOS exhibited an increased proportion of moderate OSAHS than those with mild OSAHS (38.8% vs. 29.9%). Due to the limited number of studies examining severe cases of OSAHS, it was not possible to assess the morbidity of severe OSAHS. Analysis based on BMI revealed that women diagnosed with both PCOS and obesity exhibited an elevated morbidity of OSAHS than women with PCOS but without obesity (27.5% vs. 15.8%). A subgroup analysis based on age revealed that adult women with PCOS had greater morbidity of OSAHS than adolescent women with PCOS (21.5% vs. 17.4%). Following that, a subgroup analysis based on race depicted that Caucasian woman affected by PCOS had greater morbidity of OSAHS than Asian women with PCOS (27.2% vs. 9.6%). Additionally, it was observed that studies using the Rotterdam criteria (25.4%) and studies not specifying how PCOS was diagnosed (5.6%) tended to exhibit lower morbidity of OSAHS compared to studies employing the NIH PCOS definition (31.9%) (**Table 2**).

**Table 2:** Subgroup analyses of obstructive sleep apnea in different conditions.

Subgroup Analysis of Obstructive Sleep Apnea(n)	ES (95% CI)	*P* Value	*I* ^2^(%)	*P*_h_
Overall (24)	0.208(0.147,0.276)	<0.001	98.91%	<0.001
Severity				
Mild (2)	0.299(0.251,0.348)	<0.001	0	0
Moderate (5)	0.388(0.175,0.625)	<0.001	96.02%	<0.001
Severe (1)	NA	NA	NA	NA
Not mentioned (16)	0.157(0.101,0.222)	<0.001	98.75%	<0.001
BMI				
BMI<30 (6)	0.158(0.001,0.457)	<0.001	99.28%	<0.001
BMI>30 (15)	0.275(0.156,0.411)	<0.001	97.94%	<0.001
Not mentioned (3)	0.060(0.000,0.186)	<0.001	0	<0.001
Age				
Adult (19)	0.215(0.149,0.289)	<0.001	99.08%	<0.001
Adolescent (5)	0.174(0.014,0.430)	<0.001	91.68%	<0.001
Race				
Caucasian (14)	0.272(0.101,0.485)	<0.001	98.75%	<0.001
Asian (6)	0.096(0.012,0.239)	<0.001	98.88%	<0.001
Mixed (4)	0.237(0.027,0.557)	<0.001	98.81%	<0.001
Diagnostic Criteria				
NIH (5)	0.319(0.073,0.633)	<0.001	94.97%	<0.001
Rotterdam (11)	0.254(0.142,0.384)	<0.001	97.04%	<0.001
Not mentioned (8)	0.056(0.028,0.091)	<0.001	97.22%	<0.001

ES, effect size; NA, not applicable; NIH, National Institutes of Health; P_h_, P _heterogeneity_.

### 3.5 Meta-regression analysis

**Table 3** displays the factors identified through meta-regression analysis that are correlated with morbidity estimates. We expected to identify sources of heterogeneity through meta-regression analysis, but none of the factors studied were found to be substantially correlated with the morbidity estimates in meta-regression analyses.

**Table 3:** Factors associated with OSAHS prevalence estimates identified by meta-regression analysis.

Factor	OR (95% CI)	p-value	Explained variation (%)
Age: adult (*vs.* adolescent)	0.51 (0.22 to 1.78)	0.259	11.3
BMI			8.4
< 30	1 (reference)		
> 30	0.75 (0.38 to 4.23)	0.702	
Not reported	1.02 (0.25 to 7.58)	0.852	
Ethnicity			4.2
Caucasian	1 (reference)		
Asian	0.82 (0.13 to 5.17)	0.817	
Mixed	1.15 (0.16 to 4.58)	0.724	
Diagnostic criteria			0.0
NIH definition	1 (reference)		
Rotterdam criteria	0.75 (0.16to 4.56)	0.806	
Not reported	1.12 (0.20 to 5.41)	0.859	
Severity			0.0
Mild	1 (reference)		0.0
Moderate	0.92 (0.13 to 2.27)	0.324	
Severe	1.04 (0.15 to 3.88)	0.439	
Not reported	0.82 (0.21 to 5.27)	0.628	

### 3.6 OSAHS in PCOS vs. OSAHS in non-PCOS

**Figure 3:**
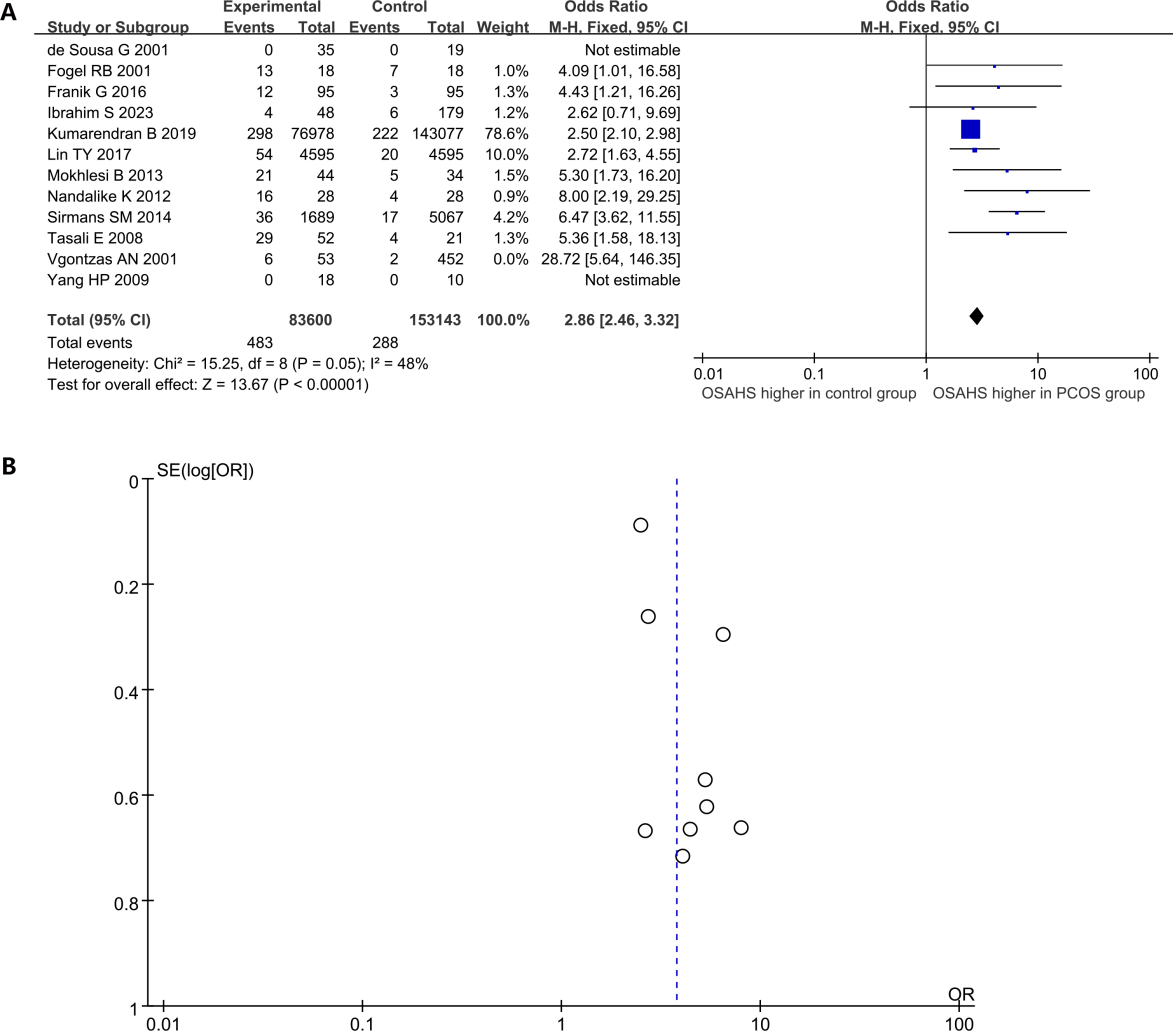
The prevalence of OSAHS in patients with PCOS compared to controls. **(A)** Forest plot of odds ratio; **(B)** Funnel plot of odds ratio.

Among women in the general population, the prevalence of OSAHS ranges from 6% to 19% (58). In the current research, 12 studies compared the morbidity of OSAHS in women with PCOS and matched controls, with the majority indicating a heightened prevalence of OSAHS among women with PCOS (**Figure 3A**). Women affected by PCOS were 2.86 times more likely to have OSAHS than controls (OR: 2.86; 95% CI: 2.46 – 3.32; 12 studies). Additionally, evidence of publication bias was found, as the funnel plot displayed asymmetry, indicating the potential existence of publication bias (**Figure 3B**). Sensitivity analysis indicated a significant reduction in heterogeneity by 15% upon excluding the study of Vgontzas AN (11), prompting its exclusion from the analysis.

### 3.7 Impact of OSAHS on clinical outcomes in women with PCOS

#### 3.7.1 Body measurement

**Figure 4:**
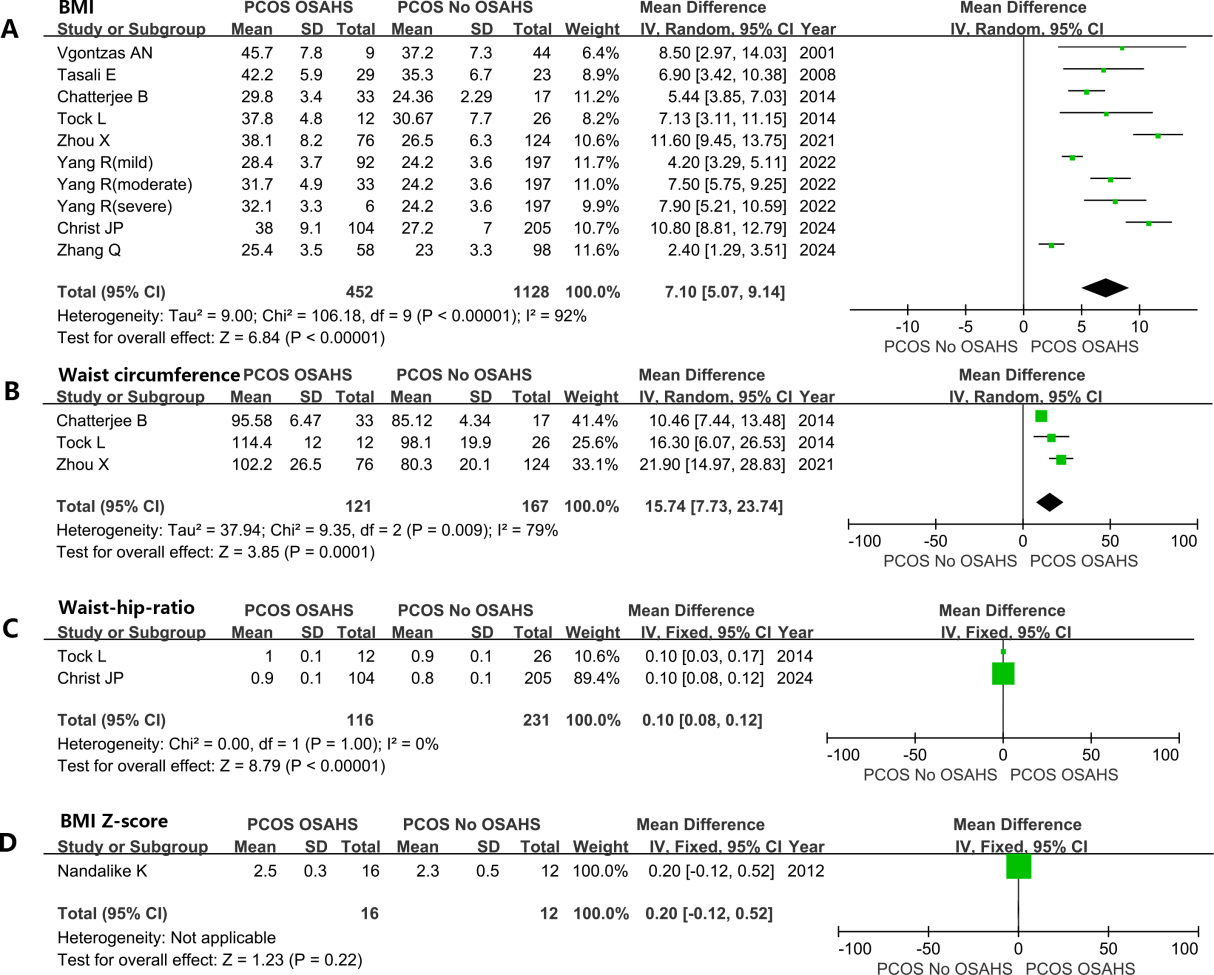
Effect of OSAHS on anthropometric measures in patients with PCOS. **(A)** BMI, **(B)** waist circumference, **(C)** Waist-hip-ratio, **(D)** BMI Z-score.

Women diagnosed with both PCOS and OSAHS have a significantly higher BMI [mean difference (MD): 7.10kg/m^2^; 95% CI: 5.07 – 9.14; I^2^ = 92%; 10 studies] (**Figure 4A**). Similarly, waistline (MD: 15.74cm; 95% CI: 7.73 – 23.74; I^2^ = 79%; 3 studies) (**Figure 4B**) and WHR (MD: 0.10; 95% CI: 0.08 – 0.12; I^2^ = 0%; 2 studies) were significantly elevated (**Figure 4C**). A study (39) reported that there was no considerable difference in BMI-Z scores between girls with and without OSAHS in a subgroup of adolescent girls with PCOS and obesity (**Figure 4D**).

#### 3.7.2 Blood pressure

Six studies depicted the impact of OSAHS on BP in women with PCOS. A meta-analysis revealed that mean systolic pressure was substantially higher in women with both PCOS and OSAHS in comparison to the women with PCOS alone, with a difference of 8.85 mmHg (95% CI: 5.16 – 12.53; I^2^ = 62%; 6 studies) (**Supplementary Figure 3A**). Similarly, diastolic pressure was significantly higher in women diagnosed with both PCOS and OSAHS in comparison to the women with PCOS but without OSAHS (MD: 4.45 mmHg; 95% CI: 2.81 – 6.08; I^2^ = 17%; 6 studies) (**Supplementary Figure 3B**).

### 3.8 Impact of OSAHS on hormonal/metabolic parameters in women affected by PCOS

#### 3.8.1 Hypertrichosis

One study (41) included in the analysis utilized the Ferriman-Gallwey (FG) Score as an indicator of hypertrichosis (**Supplementary Figure 4A**). The findings showed that this score was greater in women with both PCOS and OSAHS in comparison to the women affected by PCOS alone (MD: 1.82; 95% CI: 0.30 – 3.34).

#### 3.8.2 Sex hormone-binding globulin

Three studies included in the analysis reported SHBG levels. Women with both PCOS and OSAHS had lower SHBG levels than patients without OSAHS (MD: -22.8 nmol/L; 95% CI: -39.53 – -6.07, I^2^ = 91%) (**Supplementary Figure 4B**).

#### 3.8.3 Impact of OSAHS on testosterone in women affected by PCOS

**Figure 5:**
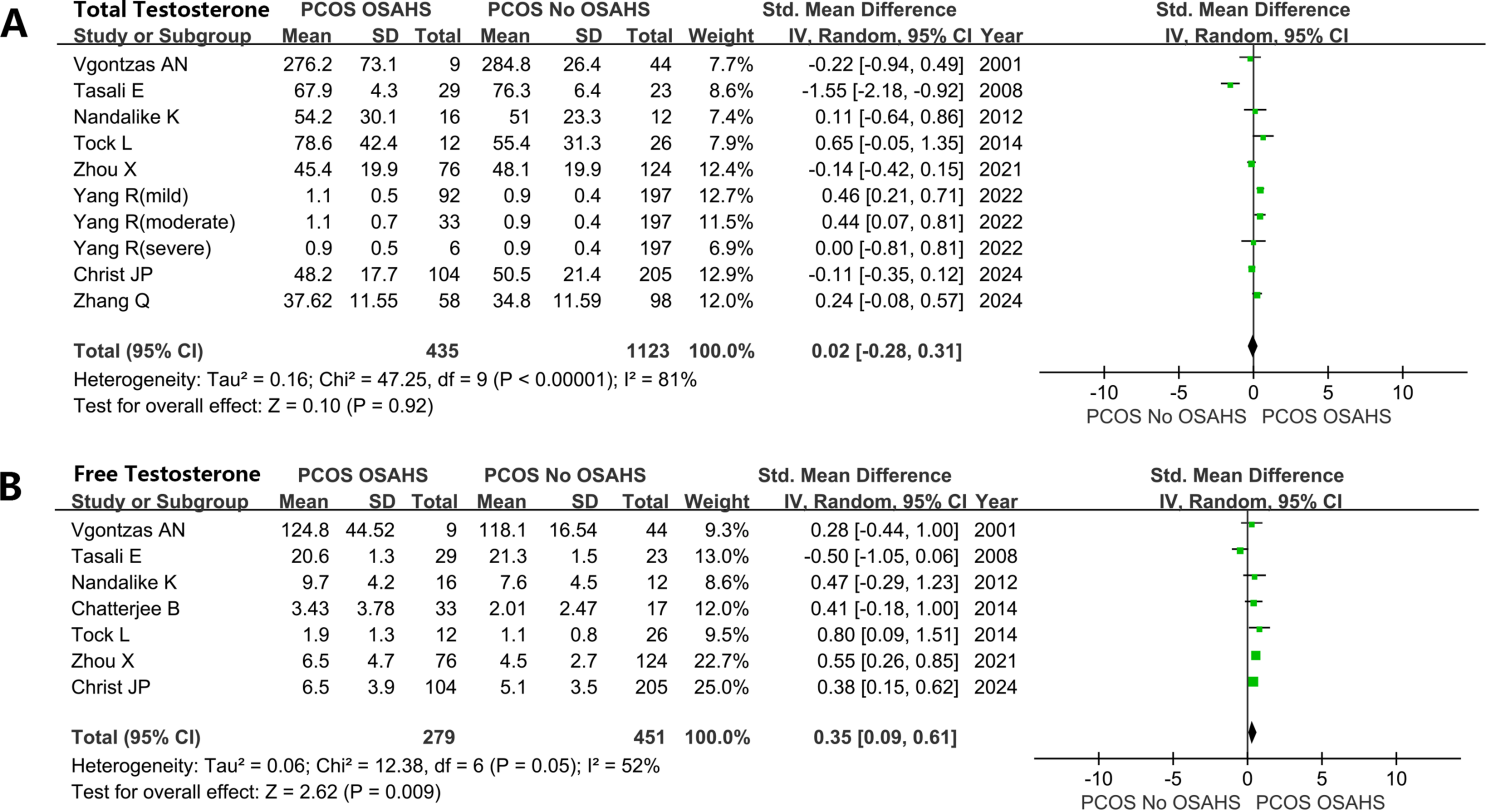
Effect of OSAHS on testosterone in patients with PCOS. **(A)** total testosterone, **(B)** free testosterone.

Women with both PCOS and OSAHS had higher testosterone: total testosterone (SMD: 0.02; 95% CI: -0.28 – 0.31; I^2^ = 81%; 10 studies) (**Figure 5A**); free testosterone (SMD: 0.35; 95% CI: 0.09 – 0.61; I^2^ = 52%; 7 studies) (**Figure 5B**).

#### 3.8.4 Glucose metabolism and IR

**Figure 6:**
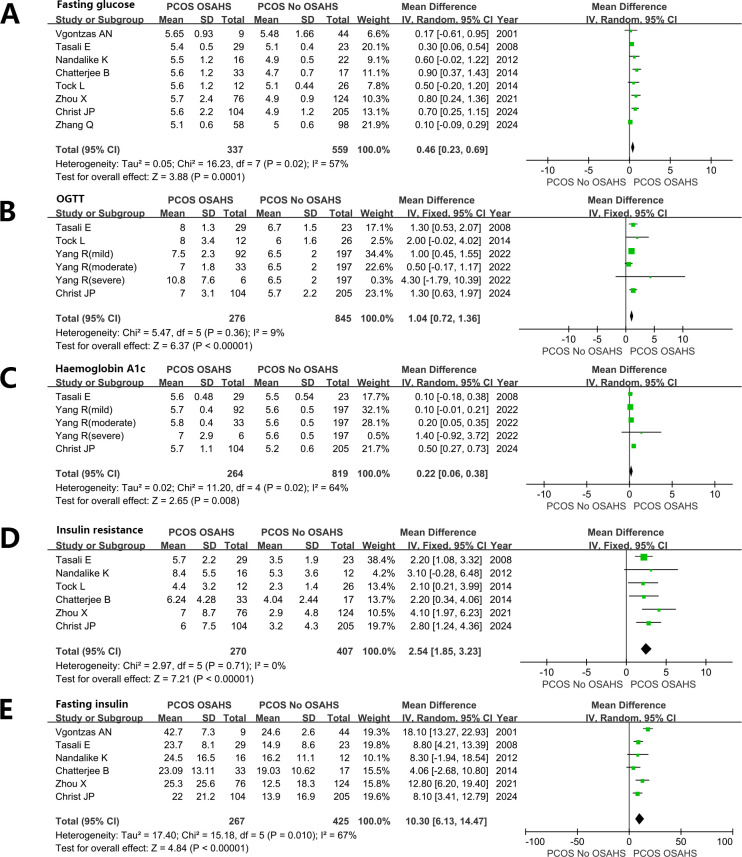
Effect of OSAHS on Glucose metabolism and insulin resistance in patients with PCOS. **(A)** Fasting plasma glucose, **(B)** 2-h OGTT, **(C)** glycosylated hemoglobin, **(D)** Homeostatic model assessment for insulin resistance, **(E)** Fasting serum insulin.

Women diagnosed with both PCOS and OSAHS exhibited elevated levels of blood glucose. Fasting plasma glucose (FPG) (MD: 0.46 mmol/L; 95% CI: 0.23 – 0.69; I^2^ = 57%; 8 studies) (**Figure 6A**). 2-h OGTT (MD: 1.04 mmol/L; 95% CI: 0.72 – 1.36; I^2^ = 9%; 6 studies) (**Figure 6B**).

glycosylated hemoglobin (MD: 0.22%; 95% CI: 0.06 – 0.38; I^2^ = 64%; 5 studies) (**Figure 6C**). Homeostatic model assessment for insulin resistance (HOMA-IR) (MD: 2.54; 95% CI: 1.85 – 3.23; I^2^ = 0%; 6 studies) (**Figure 6D**). Fasting serum INS (FINS) (MD: 10.30 μU/mL; 95% CI: 6.13 – 14.47; I^2 ^= 67%; 6 studies) (**Figure 6E**).

#### 3.8.5 Blood lipid

**Figure 7:**
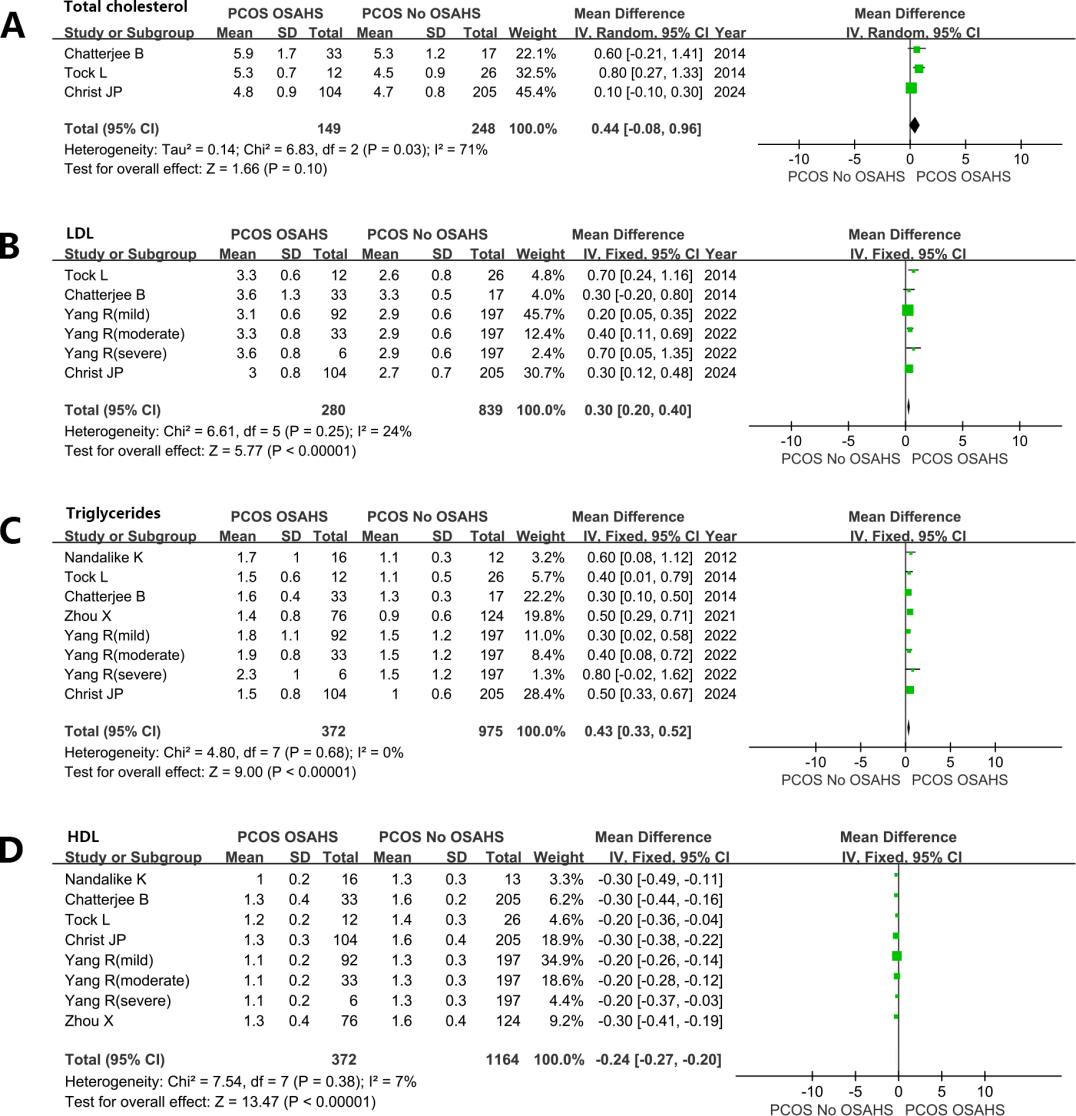
Effect of OSAHS on blood lipid in patients with PCOS. **(A)** Total cholesterol, **(B)** low-density lipoprotein, **(C)** Triglyceride, **(D)** high-density lipoprotein.

Women with PCOS, OSAHS, and obesity hypoventilation syndrome (OHS) exhibit notably elevated levels of plasma cholesterol. Total cholesterol (TC) (MD: 0.44mmol/L; 95% CI: -0.08 – 0.96; I^2 ^= 71%; 3 studies) (**Figure 7A**). low-density lipoprotein (MD: 0.30mmol/L; 95% CI: 0.20 – 0.40; I^2 ^= 24%; 6 studies) (**Figure 7B**). Triglyceride (MD: 0.43mmol/L; 95% CI: 0.33 – 0.52; I^2 ^= 0%; 8 studies) (**Figure 7C**). In contrast, plasma high-density lipoprotein (HDL) levels were lower in women diagnosed with both PCOS and OSAHS in comparison to the women affected by PCOS only (MD: -0.24 mmol/L; 95% CI: -0.27 – -0.20, I^2 ^= 7%; 8 studies) (**Figure 7D**).

## 4 Discussion

Our analysis of the available data suggested a high morbidity of OSAHS in women with PCOS. The included population was mostly Caucasians, encompassing women diagnosed with both PCOS and obesity and exhibited a significant level of selection bias. Therefore, drawing definitive conclusions about the actual morbidity of OSAHS in women with PCOS is challenging due to the limitations of the available data. The meta-analysis showed that the prevalence of OSAHS is higher in adult patients with PCOS compared to adolescent patients with PCOS, and obese patients were more likely to have OSAHS. Patients with PCOS combined with OSAHS were more prone to metabolic and androgen level disorders.

According to the current meta-analysis, nearly 21% of women with PCOS suffer from OSAHS. In a meta-analysis conducted by Kahal et al. (59), the morbidity of OSAHS in women with PCOS was as high as 35% (95% CI: 22.0% – 48.9%). The reported high morbidity of OSAHS can be explained by differences in study methodology and diverse PCOS diagnostic criteria. In the study conducted by Kahal et al. (59), the included studies encompassed conference abstracts, and the predominant diagnostic criteria utilized for PCOS were the Rotterdam criteria. Moreover, when comparing the morbidity of OSAHS in PCOS and non-PCOS groups, the meta-analysis included an article by Vgontzas (11), which yielded an odds ratio (OR) of 28.72. This particular research introduced substantial heterogeneity, a factor that significantly reduced after the exclusion of this study, as shown in **Figure 3**. The morbidity of OSAHS in PCOS derived from this study is similar to the findings of a meta-analysis by Helvaci (22%) (60). Johnsone et al. (61) employed the androgen excess mouse model of PCOS for ovarian circadian assessment and revealed that an impaired circadian clock could hamper the regulation of peripheral steroid metabolism in PCOS. Altered expression of ovarian core clock genes (Clock, Bmal1 and Per2) was found in dehydroepiandrosterone-treated PCOS mice. The intermittent hypoxia and sleep fragmentation caused by OSAHS can lead to delayed circadian rhythms and insufficient sleep. This might be the mechanism by which OSAHS affects PCOS.

Among women in the general population, the prevalence of OSAHS ranges from 6% to 19% (58). The present study proposes that women affected by PCOS are 3 times more likely to develop OSAHS than women without PCOS. However, these studies did not adequately account for important confounding factors known to influence the risk of OSAHS, encompassing age, abdominal obesity, and race. In addition, women diagnosed with PCOS were recruited from specialized clinics, whereas controls were drawn from the general population. Consequently, it remains unclear whether women with PCOS in the community face a greater risk of OSAHS than age- and obesity-matched controls. Therefore, favorable large-scale observational studies in the general population are required for it.

The findings of this study revealed a higher prevalence of OSAHS among adult women with PCOS than in adolescents. Moreover, OSAHS was more prevalent in women affected by both PCOS and obesity than in women with PCOS alone. These trends align with the increased risk of OSAHS associated with age and obesity observed in the general population (62, 63). Thus, PCOS is likely to precede the onset of OSAHS, especially in preadolescent girls in whom PCOS is a common feature (64, 65). However, in some women, OSAHS may also precede the onset of PCOS. For example, in research by Nandalike et al. (39), it was demonstrated that one-third of adolescent girls with PCOS had a history of tonsillectomy. Notably, tonsillitis and/or enlarged tonsils were identified as the primary causes of OSAHS in children (66, 67).

The limited available data propose that women with PCOS develop moderate OSAHS at a higher rate than those who develop mild OSAHS. This finding is drawn from seven studies encompassing a total of 620 participants exhibiting such characteristics. However, the sample size was insufficient to draw definite conclusions.

Obesity stands as a well-recognized risk factor associated with the onset and progression of OSAHS. Increased obesity is linked to an elevated risk of developing OSAHS (68). Thus, according to this analysis, women with both PCOS and OSAHS had higher BMI and waistline than women without OSAHS, which also suggests that women with PCOS combined with OSAHS tend to have abdominal obesity. This excessive obesity could potentially elevate the risk of type 2 diabetes and CVDs among these women. However, the precise contribution of excessive obesity or OSAHS to this increased risk has yet to be thoroughly examined. Obesity can contribute to OSAHS by causing increased parapharyngeal fat deposition, which leads to the narrowing of the upper airway. Additionally, the fat deposition in the abdomen and around the ribcage decreases thoracic compliance and reduces functional residual capacity. These factors, along with changes in neural compensatory mechanisms and the respiratory control system, further exacerbate the condition (69). Additionally, studies have demonstrated that obesity and PCOS have an inverse relationship; obesity increases PCOS prevalence while PCOS leads to weight gain and obesity (70). Obesity, PCOS and OSAHS might have a complex and controversial relationship with each other.

In addition, our meta-analysis revealed that women diagnosed with both PCOS and OSAHS exhibited higher levels of BP, resulting in higher blood lipids for atherosclerosis, compared to women without OSAHS. Although this indicates a potentially heightened risk of CVDs among women with both PCOS and OSAHS than women with PCOS alone, it is challenging to exclude the influence of obesity in this correlation based on available studies. However, in the general population, OSAHS is also correlated with heightened risks of CVDs, hypertension, and mortality (71–73). Endothelial dysfunction, increased IR, intermittent hypoxia, inflammation, sympathetic overactivity, and OS are all factors that could contribute to the emergence of cardiometabolic comorbidities in patients with OSAHS. Patel et al. (74) reported that high risk for OSAHS was an independent predictor for postoperative atrial fibrillation in patients undergoing coronary artery bypass surgery. Some researchers suggested that genetically predicted OSAHS is a potential causal risk factor for heart failure based on a large-scale population of Mendelian randomization study (75). Notably, Tasali et al. (76) treated women affected by both PCOS and OSAHS with CPAP for 8 consecutive weeks. Following this treatment, reductions were observed in diastolic pressure and morning and evening levels of norepinephrine in these patients. OSAHS may represent a significant variable risk factor in the management of women with PCOS because PCOS is correlated with an elevated risk of CVDs (77). Gao et al. (78) found that PCOS could affect cardiovascular health in women, and promote myocardial macrophage accumulation and post-myocardial infarction cardiac remodeling because of augmented splenic myelopoiesis. OSAHS and PCOS also share common pathophysiological mechanisms leading to atherosclerosis. Considering that the coexistence of OSAHS and PCOS is an independent and cumulative risk factor for cardiovascular mortality, more so than the two diseases separately, clinicians and healthcare professionals should be aware of and screen for OSAHS in patients with PCOS.

Although excess androgen is an attribute of PCOS, its involvement in the pathogenesis of OSAHS in women affected by PCOS remains controversial. Some studies have failed to establish a significant correlation between androgen levels and the severity of OSAHS (37, 79). In this study, nine studies were collectively analyzed, revealing that the levels of FT in the PCOS + OSAHS group did not exhibit substantial differences from those of the PCOS group. However, it was observed that the FT levels in the PCOS + OSAHS group were higher than those of the PCOS group. As per the free hormone hypothesis, only FT is available to interact with the androgen receptor, establishing it as the primary indicator of testosterone bioactivity (80). Furthermore, a study showed that OSAHS severity in PCOS was significantly and positively correlated with FT, even after adjusting for obesity factors (43). The modified FG Score is commonly used to assess and quantify hair growth in nine androgen-dependent regions of the body, encompassing the arms, chest, chin, lower abdomen, lower back, upper abdomen, upper back, thighs, and upper lip (81). The present study demonstrated higher FG scores in women with both PCOS and OSAHS along with lower SHBG levels in these patients. Based on the available data, a hypothesis was drawn that hyperandrogenism in women with PCOS may influence the risk of developing OSAHS. The involvement of Androgen in the pathogenesis of OSAHS is believed to occur through the mechanism of excessive androgen levels, which may be driven by increasing pharyngeal soft tissue deposition, thereby affecting respiration and increasing the risk of OSAHS. Moreover, excessive androgen is associated with impaired sensitivity and responsiveness of ventilatory chemoreceptors, potentially contributing to the higher morbidity of OSAHS in men compared to women (82, 83).

The meta-analysis suggested that IR was stronger in women with both PCOS and OSAHS than in women suffering from PCOS alone. The substantial variations in BMI observed between these two groups in the incorporated studies pose challenges in excluding obesity as a confounding factor in this correlation, even with statistical adjustments made for BMI in some of these studies (37). However, research conducted on the general population has also indicated a correlation between OSAHS and IR (84). This correlation between OSAHS and IR in women diagnosed with PCOS was further confirmed by an interventional study encompassing 19 women affected by PCOS, OSAHS, and obesity. They underwent an 8-week long CPAP treatment, resulting in a notable improvement in INS sensitivity (76). However, the reported results of these studies are based on small-sample self-controlled pre- and post-control experiments. Additionally, the risk of developing type 2 diabetes is reported to be five times higher in women with PCOS than in healthy women (85). OSAHS is additionally recognized as an independent risk factor for developing type 2 diabetes in a general population study (86). Therefore, further investigation is warranted to determine whether the primary cause of the elevated risk of type 2 diabetes development is PCOS or OSAHS. We can only hypothesize that IR is more pronounced in women affected by PCOS in the presence of significant OSAHS. However, this hypothesis needs well-conducted large cohort and interventional studies to evaluate the morbidity of type 2 diabetes and the effect of CPAP treatment on INS sensitivity and glucose metabolism in women affected by both PCOS and OSAHS.

There is a lack of studies investigating the effect of OSAHS on fertility outcomes in women diagnosed with PCOS. Since PCOS is the leading cause of anovulatory infertility (87) and OSAHS is very prevalent in women affected by both PCOS and obesity, it becomes crucial to detect whether OSAHS exerts an influence on fertility in women diagnosed with PCOS. Furthermore, there is also a lack of research examining the impact of OSAHS on mental health, depression, or anxiety in women affected by PCOS. Since both OSAHS and PCOS are independently correlated with impaired quality of life and low mood, OSAHS may exert an influence on the mental health of women with PCOS. Therefore, targeted research specifically in this domain is warranted.

Another crucial aspect of research that lacks data is race and its effect on the interaction of PCOS and OSAHS. The present study highlights a significant contrast in the morbidity of OSAHS between Asian and Caucasian individuals with PCOS, which is related to racial differences in craniofacial anatomy, fat distribution, and low arousal thresholds (88, 89). It is important to note that various facets of the clinical and metabolic profile of PCOS, such as IR, hypertrichosis, obesity, risk of type 2 diabetes, risk of CVDs, and potential response to fertility treatments, are affected by race (90, 91). Therefore, subsequent studies should analyze the metabolic and clinical characteristics of women patients from different racial backgrounds. This approach can identify high-risk populations and those particularly vulnerable to the effects of PCOS combined with OSAHS, potentially enhancing the effectiveness of interventions targeting OSAHS treatment.

CPAP is regarded as an efficient treatment for patients diagnosed with OSAHS. Observational studies have shown that patients who adhere to treatment (utilizing CPAP for >4 hours/night) experience improvements in nocturnal apnea and daytime somnolence, alongside improvements in IR, OS, and sympathetic overactivity (92, 93). Given that these mechanisms could potentially contribute to the etiology of PCOS, CPAP therapy might offer clinical benefits for women with both PCOS and OSAHS.

Notably, this study still has some limitations. Firstly, the systematic evaluation analyzed the association between OSAHS and metabolic profiles of women affected by PCOS, yet some of the included literature was at high risk of selection bias. Secondly, important confounding factors were not considered, which may lead to publication bias. Thirdly, most of the study populations were Caucasians and featured relatively small sample sizes. Thus, conclusive interpretations regarding the independent effects of OSAHS on metabolic outcomes in women affected by PCOS are difficult to make. In determining the impact of OSAHS on hyperandrogenism in women diagnosed with PCOS, the SMD analysis was applied due to the varying measures and units reported across studies. Since most studies did not report specific AHI values, it is not possible to interpret the impact of different severities of OSAHS on women with PCOS.

## 5 Conclusion

In conclusion, this meta-analysis suggests that adult women diagnosed with PCOS have a greater likelihood of suffering from OSAHS. The available results suggest that abdominal obesity, hyperandrogenism, and IR may be associated with the development of OSAHS in women affected by PCOS.

## Data Availability

The original contributions presented in the study are included in the article/**Supplementary Material**. Further inquiries can be directed to the corresponding author.

## Supplementary material

Supplementary Figure 1 Risk of bias of included studies.

Supplementary Figure 2 Funnel plot for prevalence of OSAHS in patients with PCOS.

Supplementary Figure 3 Effect of OSAHS on blood pressure in patients with PCOS. **(A)** systolic blood pressure, **(B)** diastolic blood pressure.

Supplementary Figure 4 Effect of OSAHS on Ferriman-Gallwey (FG) Score and Sex hormone-binding globulin (SHBG) in patients with PCOS. **(A)** FG, **(B)** SHBG.

## References

[1] Piltonen TT Ruokojärvi M Karro H Kujanpää L Morin-Papunen L Tapanainen JS . Awareness of polycystic ovary syndrome among obstetrician-gynecologists and endocrinologists in Northern Europe. PLoS One. (2019) 14:e0226074. doi: 10.1371/journal.pone.0226074 31877155 PMC6932801

[2] Du D Li X . The relationship between thyroiditis and polycystic ovary syndrome: a meta-analysis. Int J Clin Exp Med. (2013) 6:880–9. PMC3832324 24260593

[3] Japur CC Diez-Garcia RW de Oliveira Penaforte FR de Sá MF . Imbalance between postprandial ghrelin and insulin responses to an ad libitum meal in obese women with polycystic ovary syndrome. Reprod Sci. (2014) 21:1020–6. doi: 10.1177/1933719114522521 PMC4126217 24520086

[4] Zigarelli A Jia Z Lee H . Machine-aided self-diagnostic prediction models for polycystic ovary syndrome: observational study. JMIR Form Res. (2022) 6:e29967. doi: 10.2196/29967 35289757 PMC8965679

[5] Menon M Ramachandran V . Antithyroid peroxidase antibodies in women with polycystic ovary syndrome. J Obstet Gynaecol India. (2017) 67:61–5. doi: 10.1007/s13224-016-0914-y PMC5306098 28242970

[6] Corrie L Awasthi A Kaur J Vishwas S Gulati M Kaur IP . Interplay of gut microbiota in polycystic ovarian syndrome: Role of gut microbiota, mechanistic pathways and potential treatment strategies. Pharm (Basel). (2023) 16:197. doi: 10.3390/ph16020197 PMC9967581 37259345

[7] Legro RS Strauss JF . Molecular progress in infertility: polycystic ovary syndrome. Fertil Steril. (2002) 78:569–76. doi: 10.1016/s0015-0282(02)03275-2 12215335

[8] de Groot PC Dekkers OM Romijn JA Dieben SW Helmerhorst FM . PCOS, coronary heart disease, stroke and the influence of obesity: a systematic review and meta-analysis. Hum Reprod Update. (2011) 17:495–500. doi: 10.1093/humupd/dmr001 21335359

[9] Himelein MJ Thatcher SS . Polycystic ovary syndrome and mental health: A review. Obstet Gynecol Surv. (2006) 61:723–32. doi: 10.1097/01.ogx.0000243772.33357.84 17044949

[10] Thathapudi S Kodati V Erukkambattu J Katragadda A Addepally U Hasan Q . Anthropometric and biochemical characteristics of polycystic ovarian syndrome in South Indian women using AES-2006 criteria. Int J Endocrinol Metab. (2014) 12:e12470. doi: 10.5812/ijem.12470 24696694 PMC3968989

[11] Vgontzas AN Legro RS Bixler EO Grayev A Kales A Chrousos GP . Polycystic ovary syndrome is associated with obstructive sleep apnea and daytime sleepiness: role of insulin resistance. J Clin Endocrinol Metab. (2001) 86:517–20. doi: 10.1210/jcem.86.2.7185 11158002

[12] Gopal M Duntley S Uhles M Attarian H . The role of obesity in the increased prevalence of obstructive sleep apnea syndrome in patients with polycystic ovarian syndrome. Sleep Med. (2002) 3:401–4. doi: 10.1016/s1389-9457(02)00033-3 14592171

[13] Brassard M AinMelk Y Baillargeon JP . Basic infertility including polycystic ovary syndrome. Med Clin North Am. (2008) 92:1163–1192, xi. doi: 10.1016/j.mcna.2008.04.008 18721657

[14] Toulis KA Goulis DG Kolibianakis EM Venetis CA Tarlatzis BC Papadimas I . Risk of gestational diabetes mellitus in women with polycystic ovary syndrome: a systematic review and a meta-analysis. Fertil Steril. (2009) 92:667–77. doi: 10.1016/j.fertnstert.2008.06.045 18710713

[15] Yu HF Chen HS Rao DP Gong J . Association between polycystic ovary syndrome and the risk of pregnancy complications: A PRISMA-compliant systematic review and meta-analysis. Med (Baltimore). (2016) 95:e4863. doi: 10.1097/md.0000000000004863 PMC5181798 28002314

[16] Majidzadeh S Mirghafourvand M Farvareshi M Yavarikia P . The effect of cognitive behavioral therapy on depression and anxiety of women with polycystic ovary syndrome: a randomized controlled trial. BMC Psychiatry. (2023) 23:332. doi: 10.1186/s12888-023-04814-9 37170227 PMC10174601

[17] Taştan Bal T Akaras N Demir Ö Ugan RA . Protective effect of astaxanthin and metformin in the liver of rats in which the polycystic ovary syndrome model was formed by giving letrozole. Iran J Basic Med Sci. (2023) 26:688–94. doi: 10.22038/ijbms.2023.68032.14872 PMC10237172 37275752

[18] Moran LJ Noakes M Clifton P Buckley J Brinkworth G Thomson R . Predictors of lifestyle intervention attrition or weight loss success in women with polycystic ovary syndrome who are overweight or obese. Nutrients. (2019) 11:492. doi: 10.3390/nu11030492 30813612 PMC6470873

[19] Zhao L Zhu Z Lou H Zhu G Huang W Zhang S . Polycystic ovary syndrome (PCOS) and the risk of coronary heart disease (CHD): a meta-analysis. Oncotarget. (2016) 7:33715–21. doi: 10.18632/oncotarget.9553 PMC5085114 27220885

[20] Kahal H Tahrani AA Kyrou I Dimitriadis GK Kimani PK Barber TM . The relationship between obstructive sleep apnoea and quality of life in women with polycystic ovary syndrome: a cross-sectional study. Ther Adv Endocrinol Metab. (2020) 11:2042018820906689. doi: 10.1177/2042018820906689 32128106 PMC7036513

[21] Kumar S Anton A D'Ambrosio CM . Sex differences in obstructive sleep apnea. Clin Chest Med. (2021) 42:417–25. doi: 10.1016/j.ccm.2021.04.004 34353448

[22] LoMauro A Aliverti A . Respiratory physiology of pregnancy: Physiology masterclass. Breathe (Sheff). (2015) 11:297–301. doi: 10.1183/20734735.008615 27066123 PMC4818213

[23] Bonsignore MR Saaresranta T Riha RL . Sex differences in obstructive sleep apnoea. Eur Respir Rev. (2019) 28:190030. doi: 10.1183/16000617.0030-2019 31694839 PMC9488655

[24] Casas P Ascaso FJ Vicente E Tejero-Garcés G Adiego MI Cristóbal JA . Visual field defects and retinal nerve fiber imaging in patients with obstructive sleep apnea syndrome and in healthy controls. BMC Ophthalmol. (2018) 18:66. doi: 10.1186/s12886-018-0728-z 29499674 PMC5833149

[25] Ehrmann DA . Metabolic dysfunction in pcos: Relationship to obstructive sleep apnea. Steroids. (2012) 77:290–4. doi: 10.1016/j.steroids.2011.12.001 PMC3279609 22178788

[26] Kahal H Kyrou I Uthman O Brown A Johnson S Wall P . The association between obstructive sleep apnea and metabolic abnormalities in women with polycystic ovary syndrome: a systematic review and meta-analysis. Sleep. (2018) 41:1–12. doi: 10.1093/sleep/zsy085 29722890

[27] Fogel RB Malhotra A Pillar G Pittman SD Dunaif A White DP . Increased prevalence of obstructive sleep apnea syndrome in obese women with polycystic ovary syndrome. J Clin Endocrinol Metab. (2001) 86:1175–80. doi: 10.1210/jcem.86.3.7316 11238505

[28] Legro RS Arslanian SA Ehrmann DA Hoeger KM Murad MH Pasquali R . Diagnosis and treatment of polycystic ovary syndrome: an Endocrine Society clinical practice guideline. J Clin Endocrinol Metab. (2013) 98:4565–92. doi: 10.1210/jc.2013-2350 PMC5399492 24151290

[29] Yang HP Kang JH Su HY Tzeng CR Liu WM Huang SY . Apnea-hypopnea index in nonobese women with polycystic ovary syndrome. Int J Gynaecol Obstet. (2009) 105:226–9. doi: 10.1016/j.ijgo.2009.02.004 19345941

[30] He J Li X Yu M . The correlation of serum/plasma IGF-1 concentrations with obstructive sleep apnea hypopnea syndrome: A meta-analysis and meta-regression. Front Endocrinol (Lausanne). (2022) 13:922229. doi: 10.3389/fendo.2022.922229 36120463 PMC9471370

[31] Paczkowska K Rachoń D Berg A Rybka J Kapczyńska K Bolanowski M . Specific alteration of branched-chain amino acid profile in polycystic ovary syndrome. Biomedicines. (2023) 11:108. doi: 10.3390/biomedicines11010108 36672616 PMC9856032

[32] Lord JM Flight IH Norman RJ . Metformin in polycystic ovary syndrome: systematic review and meta-analysis. Bmj. (2003) 327:951–3. doi: 10.1136/bmj.327.7421.951 PMC259161 14576245

[33] Kim SY Park JE Lee YJ Seo HJ Sheen SS Hahn S . Testing a tool for assessing the risk of bias for nonrandomized studies showed moderate reliability and promising validity. J Clin Epidemiol. (2013) 66:408–14. doi: 10.1016/j.jclinepi.2012.09.016 23337781

[34] DerSimonian R Laird N . Meta-analysis in clinical trials. Control Clin Trials. (1986) 7:177–88. doi: 10.1016/0197-2456(86)90046-2 3802833

[35] Zhang C Li M Liu L Deng L Yulei X Zhong Y . Systemic immune-inflammation index as a novel predictor of major adverse cardiovascular events in patients undergoing percutaneous coronary intervention: a meta-analysis of cohort studies. BMC Cardiovasc Disord. (2024) 24:189. doi: 10.1186/s12872-024-03849-4 38561664 PMC10985984

[36] Higgins JP Thompson SG Deeks JJ Altman DG . Measuring inconsistency in meta-analyses. Bmj. (2003) 327:557–60. doi: 10.1136/bmj.327.7414.557 PMC192859 12958120

[37] Tasali E Van Cauter E Hoffman L Ehrmann DA . Impact of obstructive sleep apnea on insulin resistance and glucose tolerance in women with polycystic ovary syndrome. J Clin Endocrinol Metab. (2008) 93:3878–84. doi: 10.1210/jc.2008-0925 PMC2579653 18647805

[38] de Sousa G Schlüter B Menke T Trowitzsch E Andler W Reinehr T . Longitudinal analyses of polysomnographic variables, serum androgens, and parameters of glucose metabolism in obese adolescents with polycystic ovarian syndrome. Sleep Breath. (2012) 16:1139–46. doi: 10.1007/s11325-011-0620-z 22102291

[39] Nandalike K Agarwal C Strauss T Coupey SM Isasi CR Sin S . Sleep and cardiometabolic function in obese adolescent girls with polycystic ovary syndrome. Sleep Med. (2012) 13:1307–12. doi: 10.1016/j.sleep.2012.07.002 PMC3509263 22921588

[40] Mokhlesi B Scoccia B Mazzone T Sam S . Risk of obstructive sleep apnea in obese and nonobese women with polycystic ovary syndrome and healthy reproductively normal women. Fertil Steril. (2012) 97:786–91. doi: 10.1016/j.fertnstert.2011.12.024 PMC3292664 22264851

[41] Chatterjee B Suri J Suri JC Mittal P Adhikari T . Impact of sleep-disordered breathing on metabolic dysfunctions in patients with polycystic ovary syndrome. Sleep Med. (2014) 15:1547–53. doi: 10.1016/j.sleep.2014.06.023 25311833

[42] Sirmans SM Parish RC Blake S Wang X . Epidemiology and comorbidities of polycystic ovary syndrome in an indigent population. J Investig Med. (2014) 62:868–74. doi: 10.1097/01.JIM.0000446834.90599.5d 24844662

[43] Tock L Carneiro G Togeiro SM Hachul H Pereira AZ Tufik S . Obstructive sleep apnea predisposes to nonalcoholic Fatty liver disease in patients with polycystic ovary syndrome. Endocr Pract. (2014) 20:244–51. doi: 10.4158/ep12366.Or 24246334

[44] Franik G Krysta K Madej P Gimlewicz-Pięta B Oślizło B Trukawka J . Sleep disturbances in women with polycystic ovary syndrome. Gynecol Endocrinol. (2016) 32:1014–7. doi: 10.1080/09513590.2016.1196177 27348625

[45] Lin TY Lin PY Su TP Li CT Lin WC Chang WH . Risk of developing obstructive sleep apnea among women with polycystic ovarian syndrome: a nationwide longitudinal follow-up study. Sleep Med. (2017) 36:165–9. doi: 10.1016/j.sleep.2016.12.029 28599952

[46] Fernandez RC Moore VM Van Ryswyk EM Varcoe TJ Rodgers RJ March WA . Sleep disturbances in women with polycystic ovary syndrome: prevalence, pathophysiology, impact and management strategies. Nat Sci Sleep. (2018) 10:45–64. doi: 10.2147/nss.S127475 29440941 PMC5799701

[47] Kumarendran B Sumilo D O'Reilly MW Toulis KA Gokhale KM Wijeyaratne CN . Increased risk of obstructive sleep apnoea in women with polycystic ovary syndrome: a population-based cohort study. Eur J Endocrinol. (2019) 180:265–72. doi: 10.1530/eje-18-0693 PMC6410684 30763274

[48] Simon S Rahat H Carreau AM Garcia-Reyes Y Halbower A Pyle L . Poor sleep is related to metabolic syndrome severity in adolescents with PCOS and obesity. J Clin Endocrinol Metab. (2020) 105:e1827–1834. doi: 10.1210/clinem/dgz285 PMC7059992 31901092

[49] Torres-Zegarra C Sundararajan D Benson J Seagle H Witten M Walders-Abramson N . Care for adolescents with polycystic ovary syndrome: Development and prescribing patterns of a multidisciplinary clinic. J Pediatr Adolesc Gynecol. (2021) 34:617–25. doi: 10.1016/j.jpag.2021.02.002 PMC8808364 33794340

[50] Zhou X Jaswa E Pasch L Shinkai K Cedars MI Huddleston HG . Association of obstructive sleep apnea risk with depression and anxiety symptoms in women with polycystic ovary syndrome. J Clin Sleep Med. (2021) 17:2041–7. doi: 10.5664/jcsm.9372 PMC8494087 33983110

[51] Bambhroliya Z Sandrugu J Lowe M Okunlola O Raza S Osasan S . Diabetes, polycystic ovarian syndrome, obstructive sleep apnea, and obesity: A systematic review and important emerging themes. Cureus. (2022) 14:e26325. doi: 10.7759/cureus.26325 35911341 PMC9314268

[52] Underland LJ Kenigsberg Fechter L Agarwal C Sin S Punjabi N Heptulla R . Insulin sensitivity and obstructive sleep apnea in adolescents with polycystic ovary syndrome. Minerva Endocrinol (Torino). (2022). doi: 10.23736/s2724-6507.22.03619-3 35388662

[53] Yang R Gao C Yan Y Huang Y Wang J Zhang C . Analysis of the proportion and clinical characteristics of obstructive sleep apnea in women with polycystic ovary syndrome. Sleep Breath. (2022) 26:497–503. doi: 10.1007/s11325-021-02376-2 34013438

[54] Ibrahim S Mehra R Tantibhedhyangkul J Bena J Flyckt RL . Sleep and obstructive sleep apnea in women with infertility. Sleep Breath. (2023) 27:1733–42. doi: 10.1007/s11325-022-02770-4 36609819

[55] Jafar NKA Bennett CJ Moran LJ Mansfield DR . Beyond counting sheep: exploring the link between polycystic ovary syndrome and sleep health. Semin Reprod Med. (2023) 41:45–58. doi: 10.1055/s-0043-1777724 38113883

[56] Christ JP Shinkai K Corley J Pasch L Cedars MI Huddleston HG . Metabolic and endocrine status associate with obstructive sleep apnea risk among patients with polycystic ovary syndrome. J Clin Sleep Med. (2024) 20:871–7. doi: 10.5664/jcsm.11012 38217476 PMC11145041

[57] Zhang Q Wang Z Ding J Yan S Hao Y Chen H . Effect of obstructive sleep apnea on *in vitro* fertilization outcomes in women with polycystic ovary syndrome. J Clin Sleep Med. (2024) 20:31–8. doi: 10.5664/jcsm.10780 PMC10758552 37593900

[58] Senaratna CV Perret JL Lodge CJ Lowe AJ Campbell BE Matheson MC . Prevalence of obstructive sleep apnea in the general population: A systematic review. Sleep Med Rev. (2017) 34:70–81. doi: 10.1016/j.smrv.2016.07.002 27568340

[59] Kahal H Kyrou I Uthman OA Brown A Johnson S Wall PDH . The prevalence of obstructive sleep apnoea in women with polycystic ovary syndrome: a systematic review and meta-analysis. Sleep Breath. (2020) 24:339–50. doi: 10.1007/s11325-019-01835-1 PMC7127997 31111411

[60] Helvaci N Karabulut E Demir AU Yildiz BO . Polycystic ovary syndrome and the risk of obstructive sleep apnea: a meta-analysis and review of the literature. Endocr Connect. (2017) 6:437–45. doi: 10.1530/ec-17-0129 PMC5574283 28739562

[61] Johnson BS Krishna MB Padmanabhan RA Pillai SM Jayakrishnan K Laloraya M . Derailed peripheral circadian genes in polycystic ovary syndrome patients alters peripheral conversion of androgens synthesis. Hum Reprod. (2022) 37:1835–55. doi: 10.1093/humrep/deac139 35728080

[62] Wanyan P Wang J Wang W Kong Y Liang Y Liu W . Obstructive sleep apnea hypopnea syndrome: Protocol for the development of a core outcome set. Med (Baltimore). (2020) 99:e21591. doi: 10.1097/md.0000000000021591 PMC7447502 32846767

[63] Pan Z Zhuang X Li X Huang S Zhang L Lou F . Significance of vaspin in obstructive sleep apnea-hypopnea syndrome. Exp Ther Med. (2016) 11:841–5. doi: 10.3892/etm.2016.2997 PMC4774374 26998001

[64] Sir-Petermann T Codner E Maliqueo M Echiburú B Hitschfeld C Crisosto N . Increased anti-Müllerian hormone serum concentrations in prepubertal daughters of women with polycystic ovary syndrome. J Clin Endocrinol Metab. (2006) 91:3105–9. doi: 10.1210/jc.2005-2693 16720659

[65] Crisosto N Codner E Maliqueo M Echiburú B Sánchez F Cassorla F . Anti-Müllerian hormone levels in peripubertal daughters of women with polycystic ovary syndrome. J Clin Endocrinol Metab. (2007) 92:2739–43. doi: 10.1210/jc.2007-0267 17488788

[66] Reckley LK Fernandez-Salvador C Camacho M . The effect of tonsillectomy on obstructive sleep apnea: an overview of systematic reviews. Nat Sci Sleep. (2018) 10:105–10. doi: 10.2147/nss.S127816 PMC5894651 29670412

[67] Venekamp RP Hearne BJ Chandrasekharan D Blackshaw H Lim J Schilder AG . Tonsillectomy or adenotonsillectomy versus non-surgical management for obstructive sleep-disordered breathing in children. Cochrane Database Syst Rev. (2015) 2015:Cd011165. doi: 10.1002/14651858.CD011165.pub2 26465274 PMC9242010

[68] Erridge S Moussa O McIntyre C Hariri A Tolley N Kotecha B . Obstructive sleep apnea in obese patients: a UK population analysis. Obes Surg. (2021) 31:1986–93. doi: 10.1007/s11695-020-05196-7 PMC8041687 33423181

[69] Punjabi NM . The epidemiology of adult obstructive sleep apnea. Proc Am Thorac Soc. (2008) 5:136–43. doi: 10.1513/pats.200709-155MG PMC2645248 18250205

[70] Teede HJ Joham AE Paul E Moran LJ Loxton D Jolley D . Longitudinal weight gain in women identified with polycystic ovary syndrome: results of an observational study in young women. Obes (Silver Spring). (2013) 21:1526–32. doi: 10.1002/oby.20213 23818329

[71] Kartali N Daskalopoulou E Geleris P Chatzipantazi S Tziomalos K Vlachogiannis E . The effect of continuous positive airway pressure therapy on blood pressure and arterial stiffness in hypertensive patients with obstructive sleep apnea. Sleep Breath. (2014) 18:635–40. doi: 10.1007/s11325-013-0926-0 24362941

[72] Marin JM Carrizo SJ Vicente E Agusti AG . Long-term cardiovascular outcomes in men with obstructive sleep apnoea-hypopnoea with or without treatment with continuous positive airway pressure: an observational study. Lancet. (2005) 365:1046–53. doi: 10.1016/s0140-6736(05)71141-7 15781100

[73] Minoguchi K Yokoe T Tazaki T Minoguchi H Oda N Tanaka A . Silent brain infarction and platelet activation in obstructive sleep apnea. Am J Respir Crit Care Med. (2007) 175:612–7. doi: 10.1164/rccm.200608-1141OC 17341649

[74] Patel SV Gill H Shahi D Rajabalan A Patel P Sonani R . High risk for obstructive sleep apnea hypopnea syndrome predicts new onset atrial fibrillation after cardiac surgery: a retrospective analysis. Sleep Breath. (2018) 22:1117–24. doi: 10.1007/s11325-018-1645-3 PMC7778827 29460195

[75] Li Y Miao Y Zhang Q . Causal associations of obstructive sleep apnea with cardiovascular disease: a Mendelian randomization study. Sleep. (2023) 46:1–10. doi: 10.1093/sleep/zsac298 36480010

[76] Tasali E Chapotot F Leproult R Whitmore H Ehrmann DA . Treatment of obstructive sleep apnea improves cardiometabolic function in young obese women with polycystic ovary syndrome. J Clin Endocrinol Metab. (2011) 96:365–74. doi: 10.1210/jc.2010-1187 PMC3048326 21123449

[77] Guan C Zahid S Minhas AS Ouyang P Vaught A Baker VL . Polycystic ovary syndrome: a "risk-enhancing" factor for cardiovascular disease. Fertil Steril. (2022) 117:924–35. doi: 10.1016/j.fertnstert.2022.03.009 35512976

[78] Gao L Zhao Y Wu H Lin X Guo F Li J . Polycystic ovary syndrome fuels cardiovascular inflammation and aggravates ischemic cardiac injury. Circulation. (2023) 148:1958–73. doi: 10.1161/circulationaha.123.065827 PMC10713005 37937441

[79] Tasali E Van Cauter E Ehrmann DA . Relationships between sleep disordered breathing and glucose metabolism in polycystic ovary syndrome. J Clin Endocrinol Metab. (2006) 91:36–42. doi: 10.1210/jc.2005-1084 16219719

[80] Njoroge JN Tressel W Biggs ML Matsumoto AM Smith NL Rosenberg E . Circulating androgen concentrations and risk of incident heart failure in older men: The cardiovascular health study. J Am Heart Assoc. (2022) 11:e026953. doi: 10.1161/jaha.122.026953 36285783 PMC9673636

[81] Gainder S Sharma B . Update on management of polycystic ovarian syndrome for dermatologists. Indian Dermatol Online J. (2019) 10:97–105. doi: 10.4103/idoj.IDOJ_249_17 30984582 PMC6434760

[82] Lee W Nagubadi S Kryger MH Mokhlesi B . Epidemiology of obstructive sleep apnea: a population-based perspective. Expert Rev Respir Med. (2008) 2:349–64. doi: 10.1586/17476348.2.3.349 PMC2727690 19690624

[83] Kapsimalis F Kryger MH . Gender and obstructive sleep apnea syndrome, part 2: mechanisms. Sleep. (2002) 25:499–506. 12150315

[84] Tamada D Otsuki M Kashine S Hirata A Onodera T Kitamura T . Obstructive sleep apnea syndrome causes a pseudo-Cushing's state in Japanese obese patients with type 2 diabetes mellitus. Endocr J. (2013) 60:1289–94. doi: 10.1507/endocrj.ej13-0255 24047562

[85] Yau TT Ng NY Cheung LP Ma RC . Polycystic ovary syndrome: a common reproductive syndrome with long-term metabolic consequences. Hong Kong Med J. (2017) 23:622–34. doi: 10.12809/hkmj176308 29170361

[86] Tang SS Liang CH Liu YL Wei W Deng XR Shi XY . Intermittent hypoxia is involved in gut microbial dysbiosis in type 2 diabetes mellitus and obstructive sleep apnea-hypopnea syndrome. World J Gastroenterol. (2022) 28:2320–33. doi: 10.3748/wjg.v28.i21.2320 PMC9185213 35800187

[87] Balen AH Morley LC Misso M Franks S Legro RS Wijeyaratne CN . The management of anovulatory infertility in women with polycystic ovary syndrome: an analysis of the evidence to support the development of global WHO guidance. Hum Reprod Update. (2016) 22:687–708. doi: 10.1093/humupd/dmw025 27511809

[88] Tahrani AA . Ethnic differences in the pathogenesis of obstructive sleep apnoea: Exploring non-anatomical factors. Respirology. (2017) 22:847–8. doi: 10.1111/resp.13057 28417583

[89] Amin A Ali A Altaf QA Piya MK Barnett AH Raymond NT . Prevalence and associations of obstructive sleep apnea in South Asians and white Europeans with type 2 diabetes: A cross-sectional study. J Clin Sleep Med. (2017) 13:583–9. doi: 10.5664/jcsm.6548 PMC5359335 28162147

[90] Sørensen AE Wissing ML Englund AL Dalgaard LT . MicroRNA species in follicular fluid associating with polycystic ovary syndrome and related intermediary phenotypes. J Clin Endocrinol Metab. (2016) 101:1579–89. doi: 10.1210/jc.2015-3588 PMC4880172 26771704

[91] Saadeh N Alfaqih MA Mansour H Khader YS Saadeh R Al-Dwairi A . Serum homocysteine is associated with polycystic ovarian syndrome in Jordan. BioMed Rep. (2018) 9:439–45. doi: 10.3892/br.2018.1149 PMC6200971 30402228

[92] Liu C Kang W Zhang S Qiao X Yang X Zhou Z . Mandibular advancement devices prevent the adverse cardiac effects of obstructive sleep apnea-hypopnea syndrome (OSAHS). Sci Rep. (2020) 10:3394. doi: 10.1038/s41598-020-60034-1 32098974 PMC7042252

[93] Iftikhar IH Khan MF Das A Magalang UJ . Meta-analysis: continuous positive airway pressure improves insulin resistance in patients with sleep apnea without diabetes. Ann Am Thorac Soc. (2013) 10:115–20. doi: 10.1513/AnnalsATS.201209-081OC PMC3960898 23607839

