# Supplementary figures and images for: Polycystic ovary syndrome in obstructive sleep apnea-hypopnea syndrome: an updated meta-analysis
Source: Front Endocrinol (Lausanne). 2024 Aug 23;15:1418933. doi: 10.3389/fendo.2024.1418933 (PMC11377251; doi:10.3389/fendo.2024.1418933)

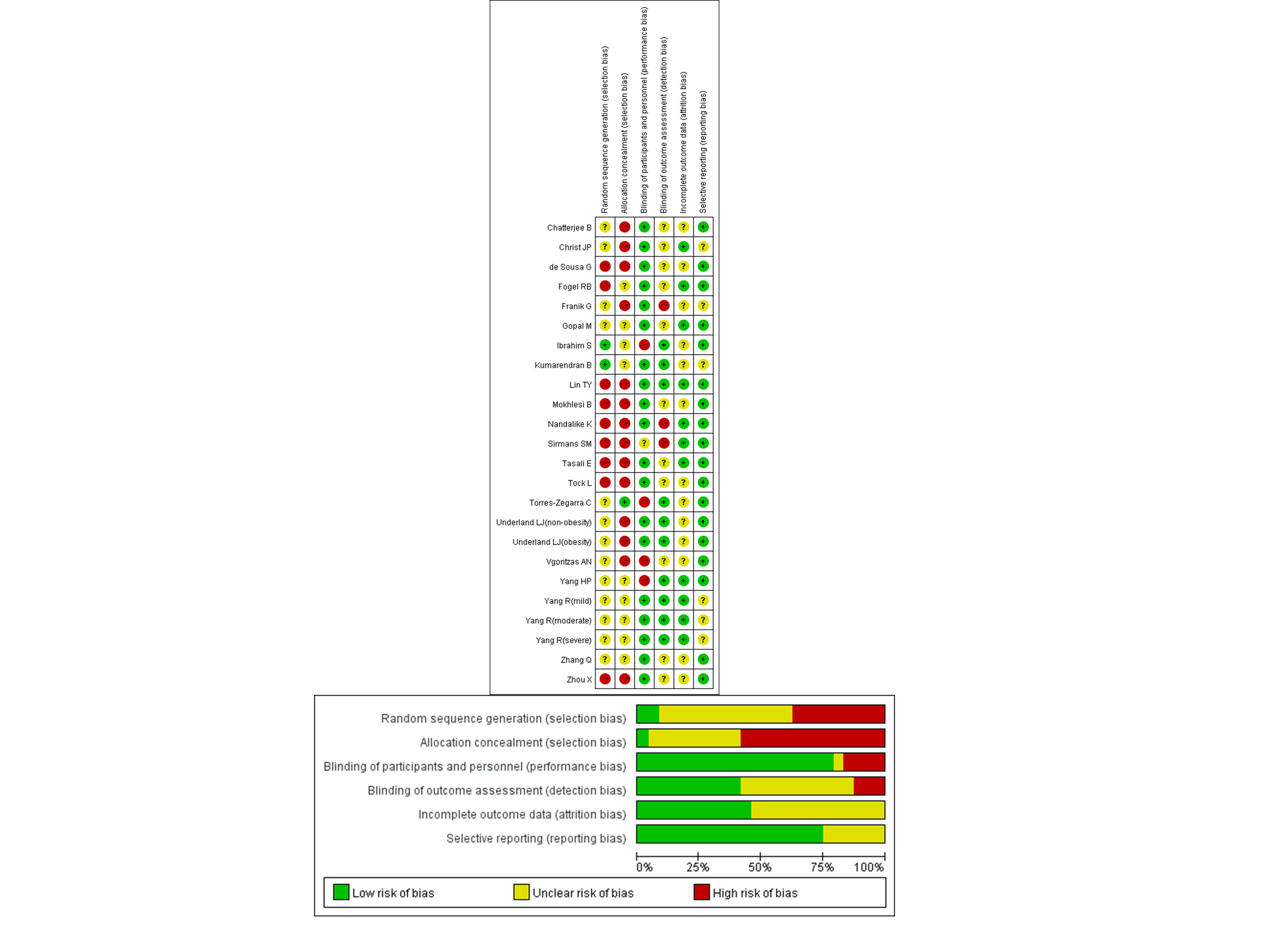

Supplement: Supplementary Figure 1 — Risk of bias of included studies. [file Image1.tif]

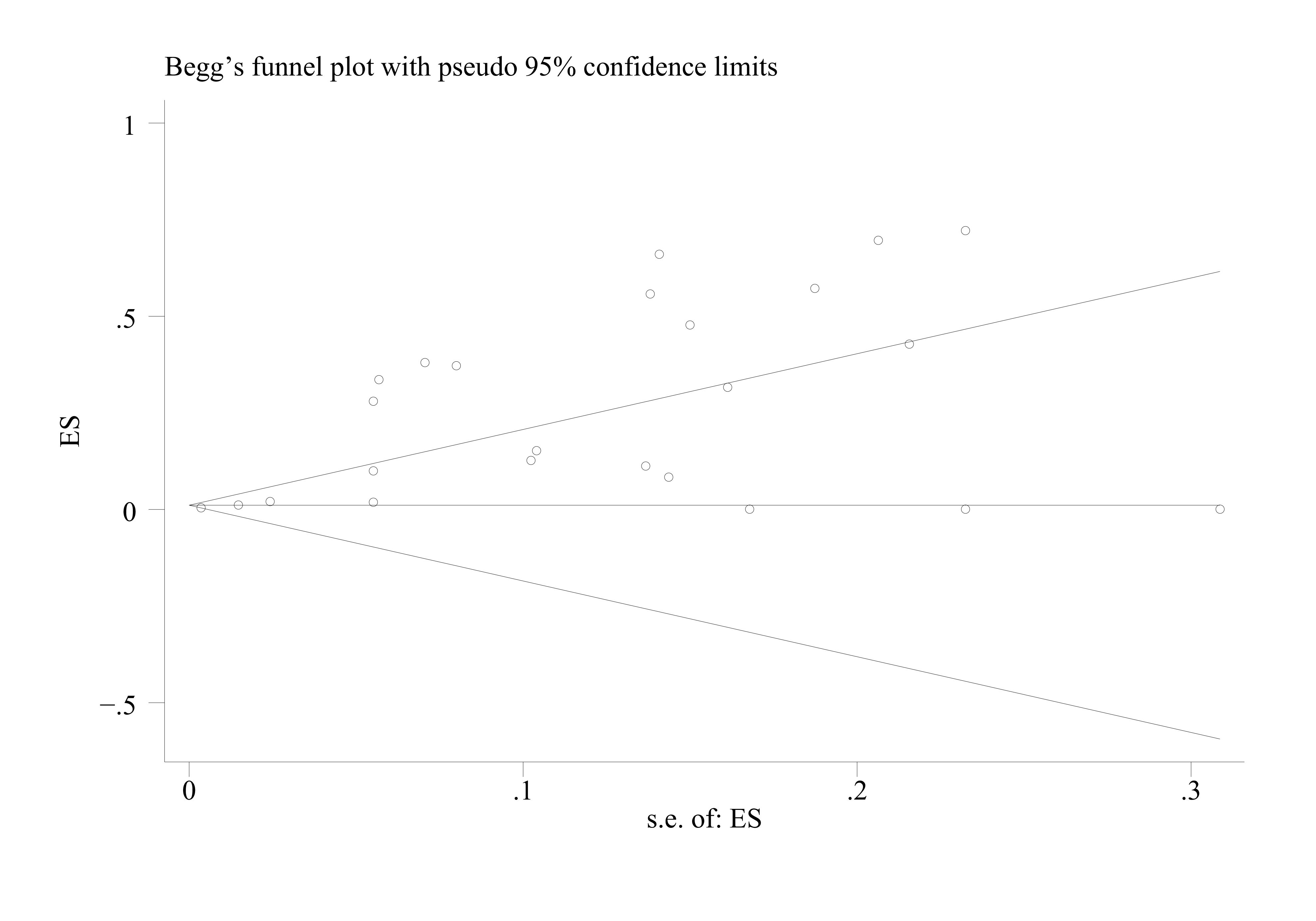

Supplement: Supplementary Figure 2 — Funnel plot for prevalence of OSAHS in patients with PCOS. [file Image2.tif]

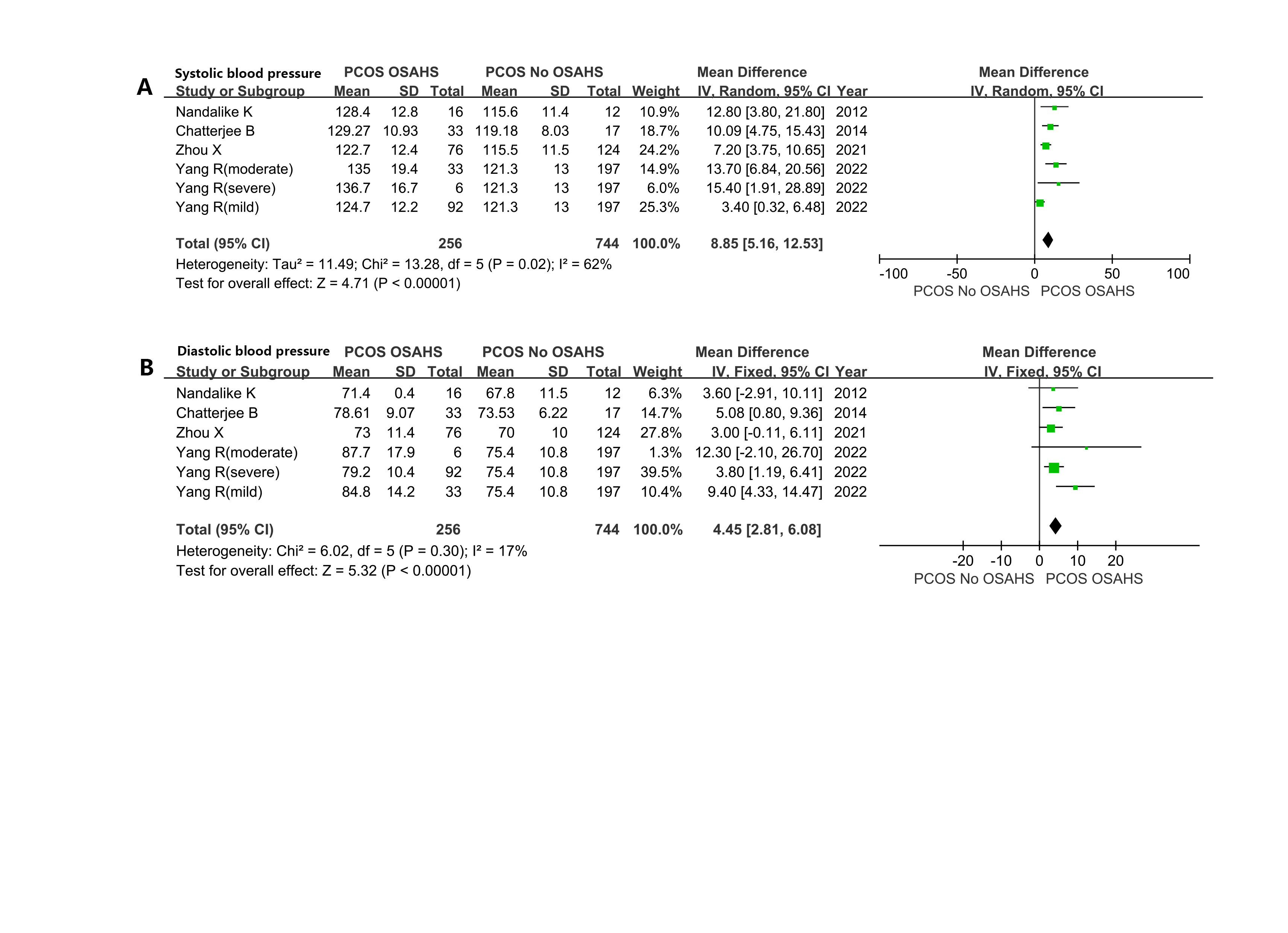

Supplement: Supplementary Figure 3 — Effect of OSAHS on blood pressure in patients with PCOS. (A) systolic blood pressure, (B) diastolic blood pressure. [file Image3.tif]

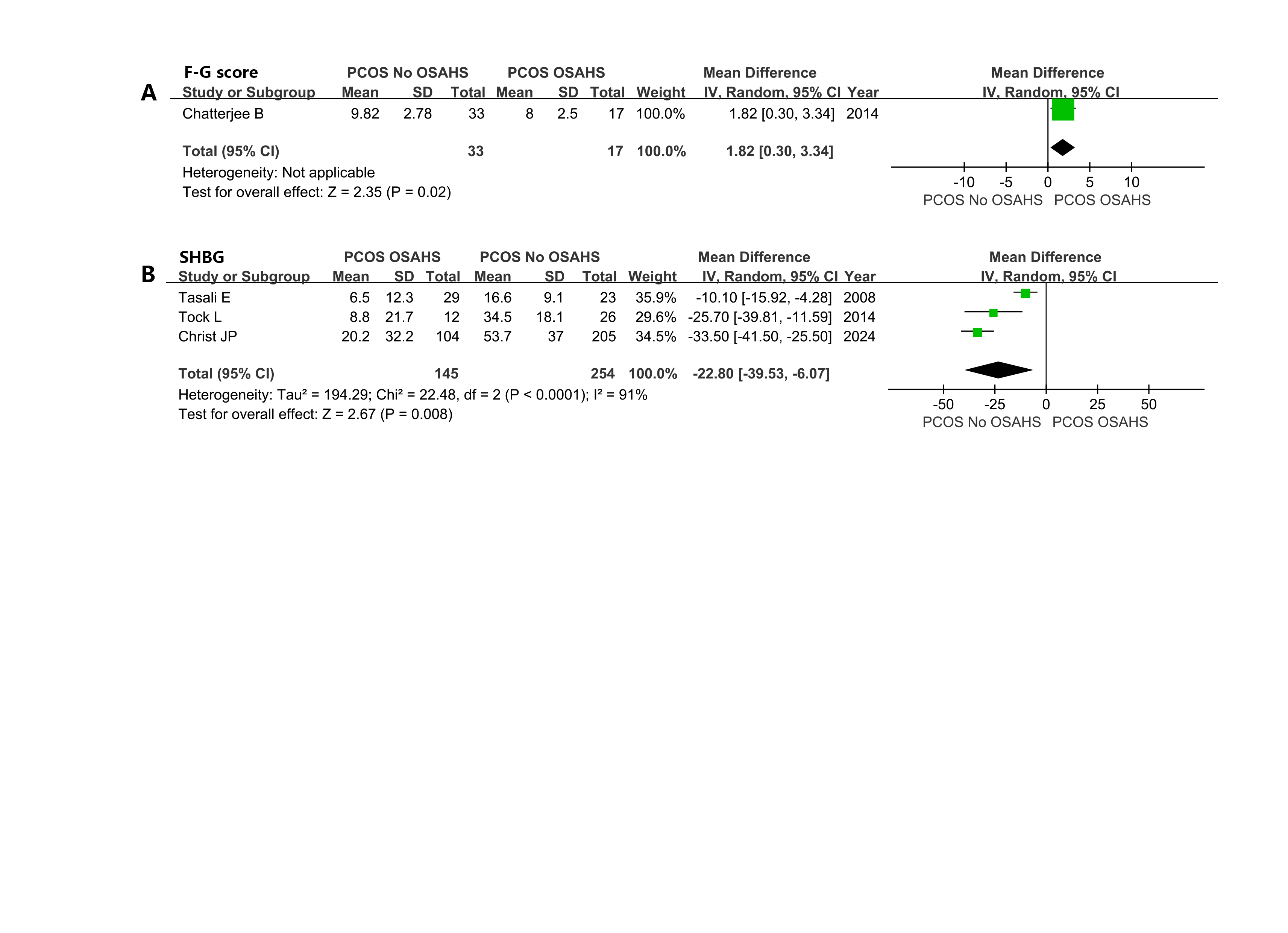

Supplement: Supplementary Figure 4 — Effect of OSAHS on Ferriman-Gallwey (FG) Score and Sex hormone-binding globulin (SHBG) in patients with PCOS. (A) FG, (B) SHBG. [file Image4.tif]
